# Complete genome sequence of biocontrol strain *Bacillus velezensis* YC89 and its biocontrol potential against sugarcane red rot

**DOI:** 10.3389/fmicb.2023.1180474

**Published:** 2023-06-02

**Authors:** Linyan Xie, Lufeng Liu, Yanju Luo, Xibing Rao, Yining Di, Han Liu, Zhenfeng Qian, Qingqing Shen, Lilian He, Fusheng Li

**Affiliations:** ^1^College of Agronomy and Biotechnology, Yunnan Agricultural University, Kunming, China; ^2^College of Resources and Environment, Yunnan Agricultural University, Kunming, China; ^3^Sugarcane Research Institute, Yunnan Agricultural University, Kunming, China

**Keywords:** *Bacillus velezensis*, biocontrol, sugarcane red rot, genome sequencing, biocontrol mechanism

## Abstract

**Introduction:**

Sugarcane is one of the most important sugar crops worldwide, however, sugarcane production is seriously limited by sugarcane red rot, a soil-borne disease caused by *Colletotrichum falcatum*. *Bacillus velezensis* YC89 was isolated from sugarcane leaves and can significantly inhibited red rot disease caused by *C. falcatum*.

**Methods:**

In this study, the genome of YC89 strain was sequenced, its genome structure and function were analyzed using various bioinformatics software, and its genome was compared with those of other homologous strains. In addition, the effectiveness of YC89 against sugarcane red rot and the evaluation of sugarcane plant growth promotion were also investigated by pot experiments.

**Results:**

Here, we present the complete genome sequence of YC89, which consists of a 3.95 Mb circular chromosome with an average GC content of 46.62%. The phylogenetic tree indicated that YC89 is closely related to *B. velezensis* GS-1. Comparative genome analysis of YC89 with other published strains (*B. velezensis* FZB42, *B. velezensis* CC09, *B. velezensis* SQR9, *B. velezensis* GS-1, and *B. amyloliquefaciens* DSM7) revealed that the strains had a part common coding sequences (CDS) in whereas 42 coding were unique of strain YC89. Whole-genome sequencing revealed 547 carbohydrate-active enzymes and identified 12 gene clusters encoding secondary metabolites. Additionally, functional analysis of the genome revealed numerous gene/gene clusters involved in plant growth promotion, antibiotic resistance, and resistance inducer synthesis. *In vitro* pot tests indicated that YC89 strain controlled sugarcane red rot and promoted the growth of sugarcane plants. Additionally, it increased the activity of enzymes involved in plant defense, such as superoxide dismutase, peroxidase, polyphenol oxidase, chitinase, and β-1,3-glucanase.

**Discussion:**

These findings will be helpful for further studies on the mechanisms of plant growth promotion and biocontrol by *B. velezensis* and provide an effective strategy for controlling red rot in sugarcane plants.

## 1. Introduction

Sugarcane (*Saccharum officinarum* L.) is one of the most important sugar crops in the world. In China, the sugarcane planting area is >85% of the sugar crop planting area, and > 92% of the total sugar is obtained from sugarcane. China is a major sugarcane planting and sugar production and marketing country (Wu et al., 2017). In 2021, the sugarcane planting area exceeded 1.301 million hm^2^, and sugarcane sugar output reached 104.4043 million tons (National Bureau of Statistics). However, the frequent occurrence of sugarcane diseases affects yield and quality, and soil-borne diseases are particularly difficult to control due to their persistence and widespread. Sugarcane red rot is a fungal disease caused by *Colletotrichum falcatum* Went.; its sexual stage is *Physalospora tucumanensis* Speg. (*Glomerella tucumanensis*) (Nithya et al., 2012). Red rot was first reported in Java, Indonesia, in 1893, representing one of the first known sugarcane diseases, leading to outbreaks in many sugarcane-producing countries (Viswanathan et al., 2009). The pathogen overwinters as hyphae, conidia, and chlamydospores in sugarcane seeds and sugarcane plants, which is the primary infection source of the disease. Pathogenic spores can be transmitted by rain, airflow, insects, and mechanical damage. In addition, they can invade plants through borer holes and growth cracks (Peñaflor and Bento, 2019). Sugarcane red rot primarily damages the stem and leaf midrib of sugarcane, which can occur from seedling stage to adult plant stage and late harvest stage. The disease in the seedling stage prevents seedlings from germinating normally, which often causes serious loss of sugarcane plants. Meanwhile, disease in the adult stage not only affects the normal growth of sugarcane plants, but also causes decay and even death (Costa et al., 2021). Indeed, red rot disease is one of the most critical sugarcane diseases in many sugarcane-growing countries, including India, Pakistan, the United States, and Bangladesh, as it can result in 25–50% sugarcane yield loss (Saksena et al., 2013) and can reduce the sugar content and purity of sugarcane juice, thus, significantly impacting the productivity of the sugarcane industry (Bukhari et al., 2012).

The main prevention and control methods for sugarcane red rot disease include disease resistance breeding, chemical control, and biological control measures. The pathogen of sugarcane red rot is prone to mutation, with more than six known variants; a disease-resistant plant becomes susceptible within 4–5 years (Sumana and Singh, 2012). Currently, the prevention and control of sugarcane red rot mainly rely on chemical fungicides. However, excessive use of chemical fungicides can lead to fungicide resistance, environmental pollution, and pose a threat to human health (Mishra and Behera, 2009). Therefore, cost-effective, chemical-free, and eco-friendly strategies for controlling sugarcane red rot are urgently required. Using bioactive agents to control plant diseases is an attractive alternative and has significant potential in the future. In fact, microorganisms with antifungal activity have been used in many crop systems. For example, *Pseudomonas* spp. (Shair et al., 2021), *Bacillus* spp. (Amna et al., 2020), and *Trichoderma* spp. (Singh et al., 2014) have been used to control sugarcane red rot in the field. Among the microbial candidates used for biocontrol, *Bacillus* species are the most useful as they can produce spores and a series of bioactive compounds that allow them to survive in adverse environmental conditions. Therefore, *Bacillus* has become one of the most studied biocontrol bacteria (Li et al., 2021; Pang et al., 2021).

Biocontrol boasts certain advantages, including environmental friendliness, safety, and the lack of induction of pesticide resistance. Microorganisms, including *Bacillus*, *Pseudomonas,* and *Paenibacillus* spp., have been used to suppress soil-borne pathogens. The main mechanisms of biological control of plant diseases include the secretion of antimicrobial substances and competition for nutrients and niches with pathogens (Backer et al., 2018; Rodriguez et al., 2019). In this way, they can induce disease resistance by promoting plant growth. In addition to these effects, many biocontrol strains increase plant resistance to pathogens via induced systemic resistance (ISR), which is triggered by a range of secondary metabolites known as elicitors (Shi et al., 2017; Gilardi et al., 2021). Different signaling pathways, such as the jasmonic acid and ethylene pathways, are activated to induce plant resistance (Ryu et al., 2004). In addition, some phenolic substances and pathogenesis-related proteins, such as chitinase, β-l,3-glucanase (β-1,3-GA), peroxidase (POD), polyphenol oxidase (PPO), superoxide dismutase (SOD), and phenylalanine aminolysis, are involved in plant disease resistance.

*Bacillus velezensis* has been widely used in agricultural production owing to its environmental safety, straightforward industrial production, and good biocontrol efficacy (Sun et al., 2022). *B. velezensis* YC89 is a sugarcane endophyte isolated from sugarcane leaves and can inhibit 78% of the etiological pathogens of sugarcane red rot (Xie et al., 2020). However, the relative efficacy of *B. velezensis* YC89 against sugarcane red rot and its biological control mechanism remains unknown. The current study sought to investigate the mechanisms underlying the biocontrol ability of *B. velezensis* YC89 and determine its complete genome sequence. To this end, a greenhouse experiment was performed. Results revealed *B. velezensis* YC89 could serve as a potential biocontrol agent of sugarcane red rot and promote sugarcane plant growth. To understand the molecular mechanism involved in the plant–microbe interaction, high-quality genome assembly and annotation of *B. velezensis* YC89 was performed. Collectively, the results of the current study support the application of *B. velezensis* to sugarcane plants in the field to protect against red rot disease.

## 2. Materials and methods

### 2.1. Bacterial and fungal strains and growth conditions

The fungus *C. falcatum* Went., antagonistic bacterium *B. velezensis* YC89, and an unknown strain X22 used in this study were isolated from the leaves of infected sugarcane plants and healthy plants, respectively. *B. velezensis* YC89 was deposited as a reference strain (strain N°60,902) at the China General Microbiological Culture Collection Center. All bacteria were cultured on Luria-Bertani agar media and incubated at 28°C for 24 h. *C. falcatum* Went. was cultivated on a potato dextrose agar (PDA) at 28°C for 7 d.

### 2.2. DNA extraction, genome sequencing, and annotation

The genomic DNA of *B. velezensis* YC89 was extracted using the HiPure Bacterial DNA kit (Magen, Guangzhou, China) according to the manufacturer’s instructions. The quality of the extracted DNA was determined using Qubit (Thermo Fisher Scientific, Waltham, MA) and Nanodrop (Thermo Fisher Scientific, Waltham, MA) for the corresponding assays.

The whole genome was sequenced using the Pacific Biosciences platform (PacBio, Menlo Park, CA) and Illumina Miseq platform at Genedenovo Biotechnology (Guangzhou, China). Qualified genomic DNA was fragmented with G-tubes (Covaris, Woburn, MA, United States) and end-repaired to prepare SMRTbell DNA template libraries (with fragment size of >10 Kb selected by blue pippin system) according to the manufacturer’s specification (PacBio, Menlo Park, CA). Liraray quality was detected by Qubit® 2.0 Flurometer (Life Technologies, CA, United States) and average fragment size was estimated on a Bioanalyzer 2,100 (Agilent, Santa Clara, CA). SMRT sequencing was performed on the Pacific Biosciences Sequel (PacBio, Menlo Park, CA) according to standard protocols. The PacBio reads were *de novo* assembled using Microbial Assembly (smrtlink8), HGAP4, and Canu (v.1.6) software. The depth of genome coverage was analyzed using the pbalign (BLASR, v.0.4.1) tool. The complete genome sequence of *B. velezensis* YC89 was deposited in GenBank under the accession number CP0392499.

The genes were annotated by alignment with those deposited in diverse protein databases, including the NCBI non-redundant (NR) protein sequence database, UniProt/Swiss-Prot, Kyoto Encyclopedia of Genes and Genomes (KEGG), gene ontology (GO), and Cluster of Orthologous Groups of proteins (COG). Protein family annotation was performed using Pfam_Scan (version 1.6) based on the Pfam database (version 32.0) (Finn et al., 2014). Gene sequences annotated from the YC89 genome were basic local alignment search tool (BLAST)ed. using the carbohydrate-active enzyme (CAZyme) database.

### 2.3. Phylogenetic tree

To determine the taxonomic position of YC89 in the genus *Bacillus*, sequence analysis of YC89 and 30 other *Bacillus* strains was performed based on the 16S rDNA gene, and an evolutionary tree was constructed. Genome-wide comparative analysis was then performed for YC89 and *B. velezensis* FZB42, *B. velezensis* CC09, *B. velezensis* SQR9, *B. velezensis* GS-1, and *B. amyloliquefaciens* DSM7, and genes that were single copies in the whole genome were selected. The evolutionary tree was constructed using iqtree (version 1.6.3) (Lam-Tung et al., 2015).

### 2.4. Gene family analysis

All genomes used in this study were downloaded in the FASTA format from the NCBI database. Basic information on the reference strains is provided in Supplementary Table S1. For comparative analyses of the orthologous and exclusive genes between YC89 and the other closest genomes, the amino acid (or nucleotide) sequences of *B. velezensis* FZB42, *B. velezensis* CC09, *B. velezensis* SQR9, *B. velezensis* GS-1, and *B. amyloliquefaciens* DSM7 were filtered in FASTA format to remove low-quality sequences based on the length and percentage of stop codons. Diamond (version 2.0.7) was used to compare the amino acid (or nucleotide) sequences of all species investigated in the study (Buchfink et al., 2021). OrthoMCL (version 1.4) was used for similarity clustering, a list of homologous genes grouped into clusters was obtained, and the species distribution of each protein cluster was determined (Li et al., 2003). Subsequently, the Mauve software (version 2.3.1) was employed to analyze the genome collinearity of six *Bacillus* strains (FZB42, CC09, SQR9, GS-1, DSM7, YC89) (Darling et al., 2004).

Based on collinearity, Pyani was used to calculate the average nucleotide identity (ANI) of the pairwise genome alignment region between the target genome and closely related reference genome (Pritchard et al., 2016). The ANI value is an index for comparing two genomic relatives at the nucleotide level and is defined as the average base similarity between homologous segments of two microbial genomes, which is characterized by a high degree of discrimination between closely related species. Usually, an ANI value of 95% is set as the classification threshold to distinguish species.

### 2.5. Secondary metabolic genes and mining for genes related to beneficial plant traits

The genome of *B. velezensis* YC89 was analyzed using antiSMASH 4.0 (version 4.1.0) with web server1 to predict putative secondary metabolites (Blin et al., 2017). Functional genes involved in plant growth promotion, such as those responsible for indole acetic acid (IAA) production, phosphate solubilization, nitrogen fixation, and genes related to the induction of resistance substance synthesis, were searched in the KEGG database according to the methods described by Kumar et al. (2019) and Shi et al. (2017), and gene similarity was compared using BLAST.

### 2.6. Analysis of the biocontrol characteristics

#### 2.6.1. Siderophore production test

The ability of the YC89 strain to produce siderophores was determined according to the method of Schwyn and Neilands (1987). The activated strain was inoculated on Chrome Azurol S (CAS) medium and incubated at 28°C for 7 d to observe whether a yellow transparent aperture was produced. All experiments were performed in triplicate.

#### 2.6.2. Detection of cellulase activity

Carboxymethyl cellulose agar (Zheng et al., 2011) was used to detect cellulase activity. The strains were inoculated in carboxymethyl cellulase identification medium and incubated at 28°C for 3 d. The plates were then dyed with Congo red (1 mg/mL) for 15 min and rinsed with NaCl (1 mol/L) for several minutes (Guo et al., 2016). The plates were then examined to determine whether cellulose hydrolysis circle was produced around the colonies; the presence of a cellulose hydrolysis circle indicates that the strain can produce carboxymethyl cellulase. All experiments were performed in triplicate.

#### 2.6.3. Phosphate solubilization assay

The strains were inoculated on a solid phosphate solubilization medium with tricalcium phosphate and calcium phytate as substrates (Kumar et al., 2013). The formation of transparent halo zones around the bacterial colonies after 7 d of incubation at 28°C was considered an indication of phosphate solubilizing activity. All experiments were performed in triplicate.

### 2.7. Biocontrol effect of YC89 on sugarcane red rot in the greenhouse

#### 2.7.1. Bacterial inoculation and treatments

One single colony on the medium was picked and inoculated into Luria-Bertani broth and incubated at 28°C for 24 h under shaking conditions (180 rpm). The fermented liquid was centrifuged at 10,000 rpm for 10 min, and the supernatant was discarded, suspended in sterile water, and adjusted to a concentration of 1 × 10^8^ CFU/mL for further experiments.

#### 2.7.2. Preparation of the pathogen spore suspension

*Colletotrichum falcatum* Went. was inoculated onto solid PDA medium and incubated at 28°C for 7 d. A sterile punch (*d* = 5 mm) was used to remove a plug from the edge of the pathogen colony and transfer it to the potato liquid culture solution. The culture was then incubated for 7 d at 28°C with shaking at 180 rpm, and filtered through a double layer of sterile gauze to obtain a suspension of pathogen spores. The spores were enumerated under a microscope with a hemocytometer plate, and diluted with sterile water to a concentration of 1 × 10^7^ conidia/mL.

#### 2.7.3. Experimental design

For the greenhouse experiment, the sugarcane cultivar (ROC 22) was the test plant. Two sugarcane endophytes, YC89 and X22, were used for the pot experiments. Five treatments were applied: (1) water (control), (2) carbendazim (1 mg/mL, positive control), (3) YC89, (4) X22, and (5) YC89 + X22. All experiments had six replicates, with 12 seedlings per treatment. From seedling emergence to six leaves, the plants in treatment groups 3, 4, and 5 were treated with three applications of bacterial suspension, while group 2 was treated twice with carbendazim. Water was applied as a control. At 15 d after the final bacterial treatment, the seedlings in each treatment group were inoculated with 1 L of *C. falcatum* spore suspension (1 × 10^7^ conidia/mL).

#### 2.7.4. Sample collection

After inoculation with *C. falcatum*, sugarcane leaves were collected on Days 0, 1, 3, 5, and 7 to detect the changes in related defense enzyme activities in sugarcane at different time points. The leaves were stored in a deep freezer at −80°C for further biochemical analysis.

Disease incidence and severity were recorded within 7–10 d of the appearance of the first symptoms. Disease severity was rated on a scale from 0 to 5 according to the symptoms of plant disease in each plant: 0, no symptoms; 1, appearance of small red spots; 2, enlargement of red spots; 3, spots extend along the midrib to form strips of red to brown parallel to the vein; 4, breakage of blade; 5, spear leaf and growth point rot to death. The disease index and disease control effects were calculated as follows:

Disease index = [∑(The number of diseased plants in this grade × Disease grade)/(Total number of plants investigated × the highest disease grade)] × 100.

Control efficacy (%) = [(Disease index of control – Disease index of treated group)/Disease index of control] × 100.

#### 2.7.5. Analysis of plant defense-related enzymes

The activities of plant defense-related enzymes (SOD, PPO, chitinase, and β-1,3-GA) were assessed during the process of *B. velezensis* YC89-triggered ISR against *C. falcatum* in sugarcane. The activities of SOD, PPO, chitinase, and β-1,3-GA in plant leaves were measured using the corresponding test kits as described in the study by Jiang et al. (2019) (Nanjing Jiancheng Biological Engineering Institute, Nanjing, China), according to the manufacturer’s instructions.

### 2.8. Statistical analyses

All data were analyzed by analysis of variance in IBM SPSS Statistics 25 (IBM Corp., Armonk, NY, United States). Differences between means were compared using the LSD test (Fisher’s protected least significant difference test) at *p* = 0.05. *p* < 0.05 was considered statistically significant.

## 3. Results

### 3.1. Genomic features and annotation of *Bacillus velezensis* YC89

**Figure 1:**
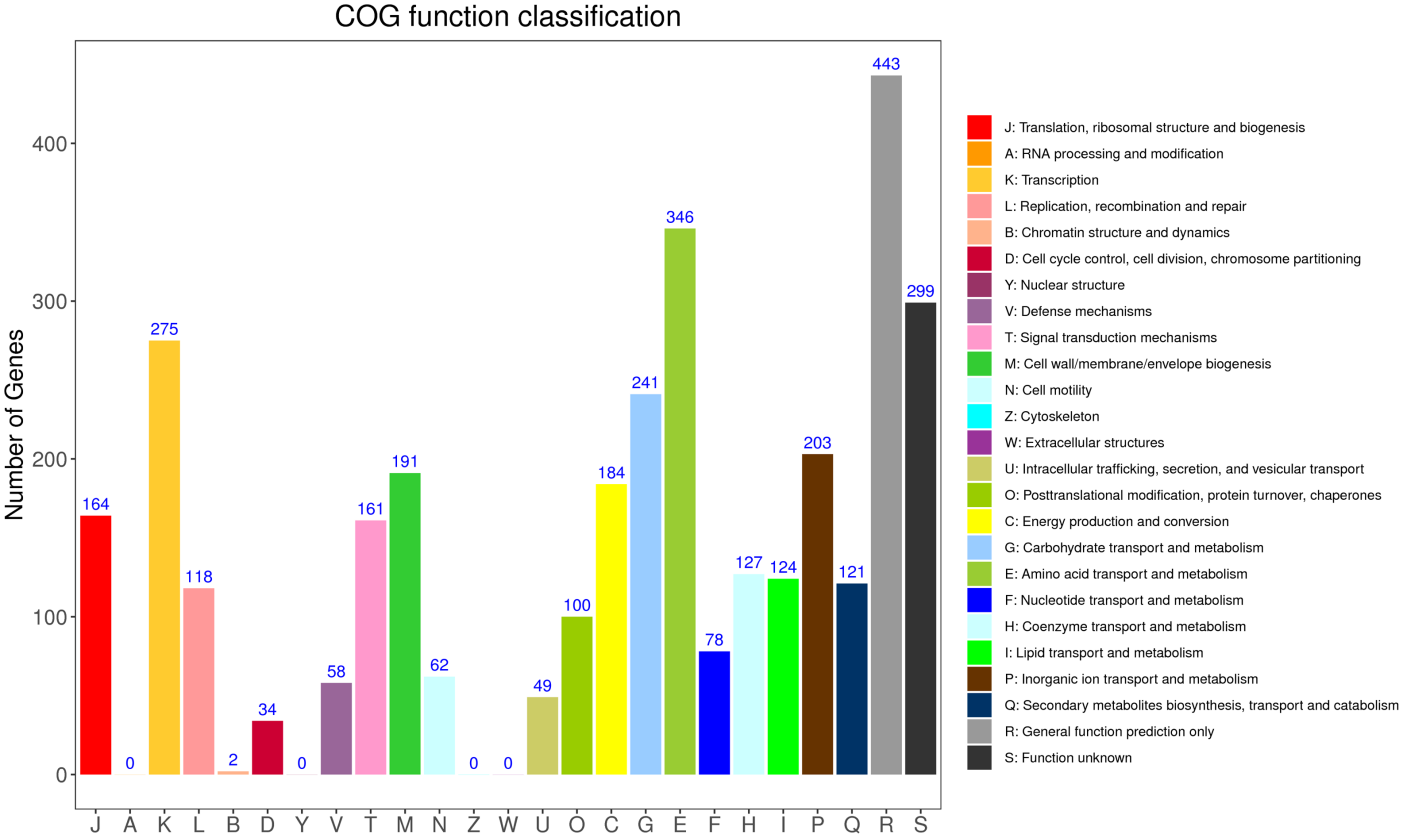
Circular genome map of *Bacillus velezensis* YC89. From the innermost to the outermost: ring 1 GC skew positive (Orange) and negative (purple); ring 2 for GC content, Orange is greater than the average, blue is less than the average; ring 3 for distribution of rRNAs (red) and tRNAs (black); ring 4 COG classifications of protein-coding genes on the reverse strand; ring 5 COG classifications of protein-coding genes on the forward strand; ring 6 for genome size (black line).

The complete genome sequence of *B. velezensis* YC89 was composed of a 3.95 Mb circular chromosome that included 3,711 coding sequences (CDS) with an average length of 922.05 bp and an average G + C content of 46.62% (Figure 1). The chromosome contained 27 rRNAs, 86 tRNAs, and two genomic islands, with an average length of 39.39 kb. Among the 3,711 CDS, 3698 were annotated functionally, and the remaining 13 were hypothetical. Of the predicted genes, 3,696 (99.60%), 3,380 (91.08%), 2,785 (75.05%), and 2,278 (61.38%) matched in the NR, Swiss-Prot, COG, and KEGG databases, respectively. The features of the YC89 genome and a comparison with other closely related *Bacillus* genomes are presented in Table 1. In comparison, the entire genome size of the five *B. velezensis* strains ranged from 3.92–4.16 Mb, the G + C content ranged from 46.1–47.06%, and the predicted coding genes ranged from 3,711–4,187. The YC89 strain and the other five strains contained one circular chromosome without a plasmid. The genome size and protein-coding genes of strains YC89 and FZB42 were smaller than those of the other strains.

**Table 1:** Genomic features of *Bacillus velezensis* YC89 and its close-related strains.

Features	*B. velezensis* YC89	*B. velezensis* GS-1	*Bacillus velezensis* FZB42	*B. velezensis* CC09	*B. velezensis* SQR9	*B. amyloliquefaciens* DSM7
Genome size (bp)	3,953,810	4,030,799	3,918,589	4,167,153	4,117,023	3,980,199
G + C content (%)	46.62	47.06	46.5	46.1	46.1	46.1
Protein-coding genes	3,711	4,187	3,893	4,141	4,096	4,135
tRNA	86	86	88	73	72	24
rRNA	27	27	29	24	21	30

**Figure 2:**
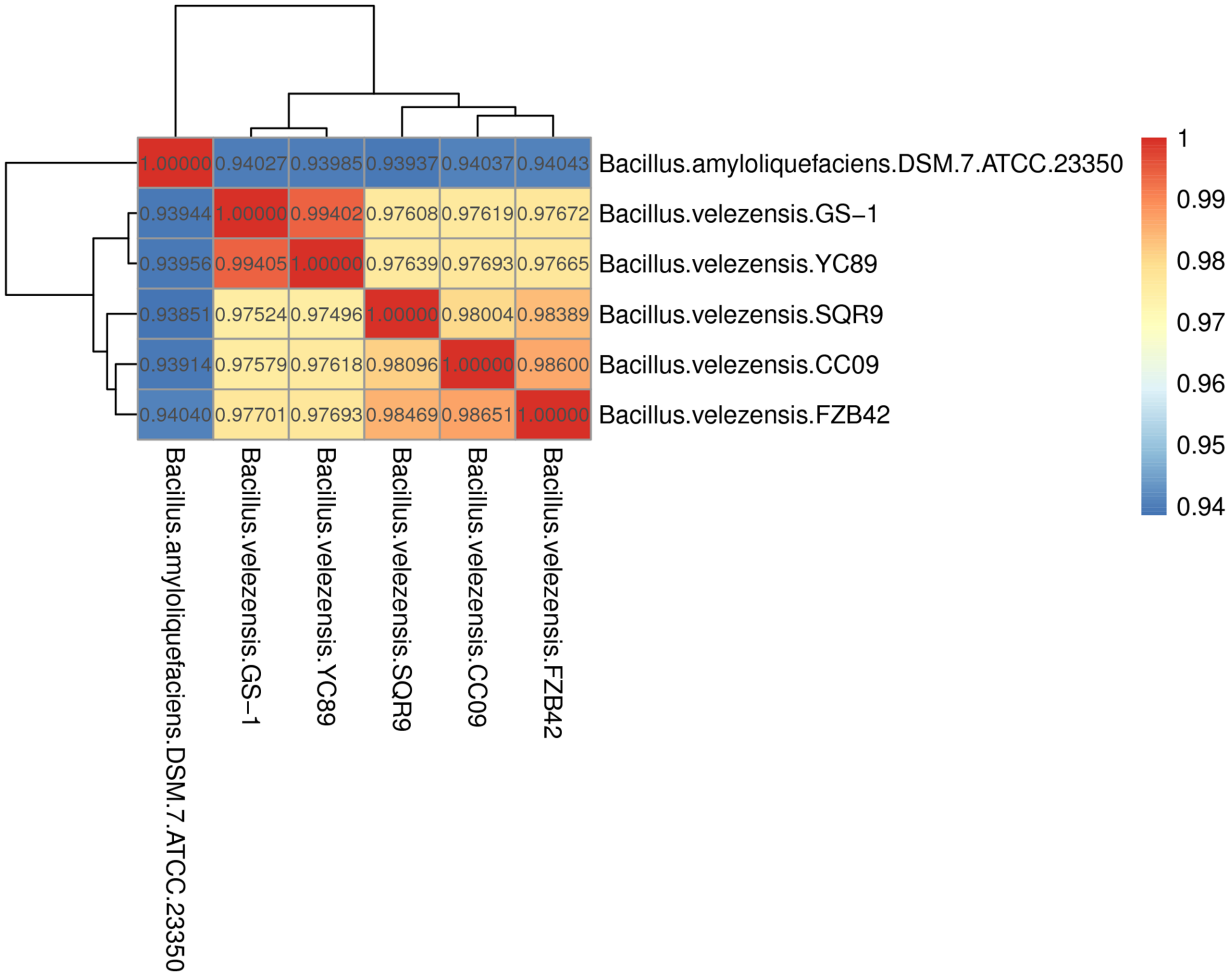
Distribution of genes across COG functional categories in the chromosome of YC89.

According to GO annotation, the genes were classified into 59 functional groups, and the genes involved in biological processes were the most abundant (Supplementary Figure S1). Among the biological process groups, the number of genes related to cellular processes was the highest (2708) (Supplementary Table S2). Based on the COG database, 3,380 genes were assigned to 21 COG categories (Figure 2). The general function prediction-only category represented the largest group (443 genes, 11.94% of all CDS), followed by amino acid transport and metabolism (346 genes, 9.32% of all CDS). According to KEGG annotation, 2,172 genes (58.52% of all CDS) were assigned to 23 KEGG pathways, and the largest number of identified genes were classified into metabolic pathways. Among these pathways, the most represented pathways included carbohydrate metabolism (242 genes, 6.52% of all CDS), followed by amino acid metabolism, and metabolism of cofactors and vitamin pathways (Supplementary Figure S3).

### 3.2. Identification of strain YC89

These results indicate that YC89 is closely related to *B. velezensis* FZB42 and *B. amyloliquefaciens* 6B (Supplementary Figure S3). For an in-depth analysis of the taxonomic status of YC89 strain, a genome-wide phylogenomic tree was constructed. The results revealed that strain YC89 formed a close genetic relationship with strain *B. velezensis* GS-1 (Supplementary Figure S4).

**Figure 3:**
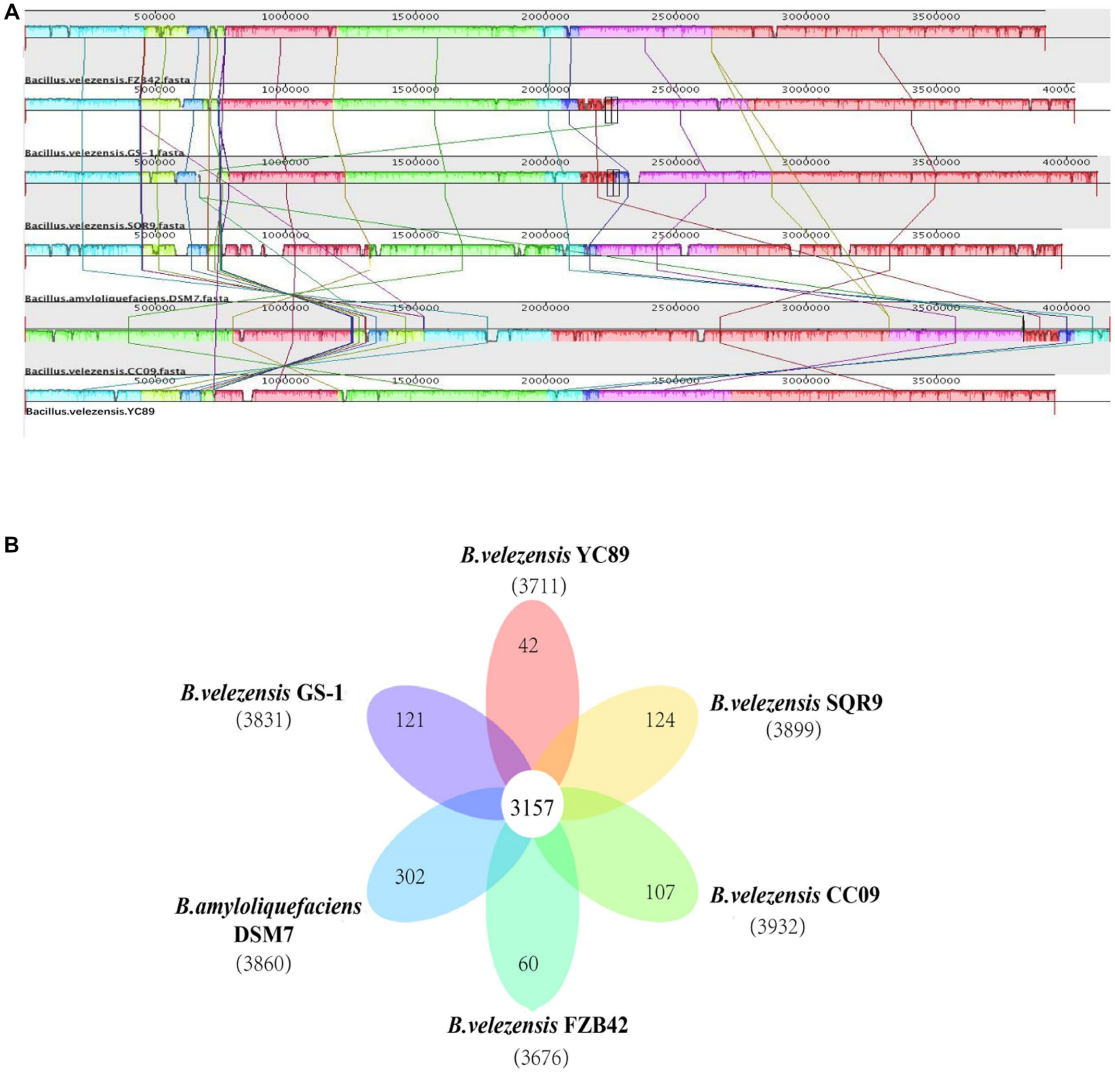
Heatmap of pairwise average nucleotide identity (ANI) values for whole genomes of YC89 and five other *Bacillus* species.

ANI is a powerful approach for assessing the evolutionary distance among bacterial species based on digital whole-genome comparisons, with values closer to 1 indicating higher similarity. Based on ANI values, the genome sequence of YC89 displayed the highest similarity with *B. velezensis* with ANI values >97%; however, the ANI values between YC89 and *B. amyloliquefaciens* were < 95% (Figure 3). Strains being compared having ANI values >96% are typically regarded as the same species. Therefore, YC89 does not belong to *B. amyloliquefaciens*.

### 3.3. Comparison of YC89 with *Bacillus* spp. strains

**Figure 4:**
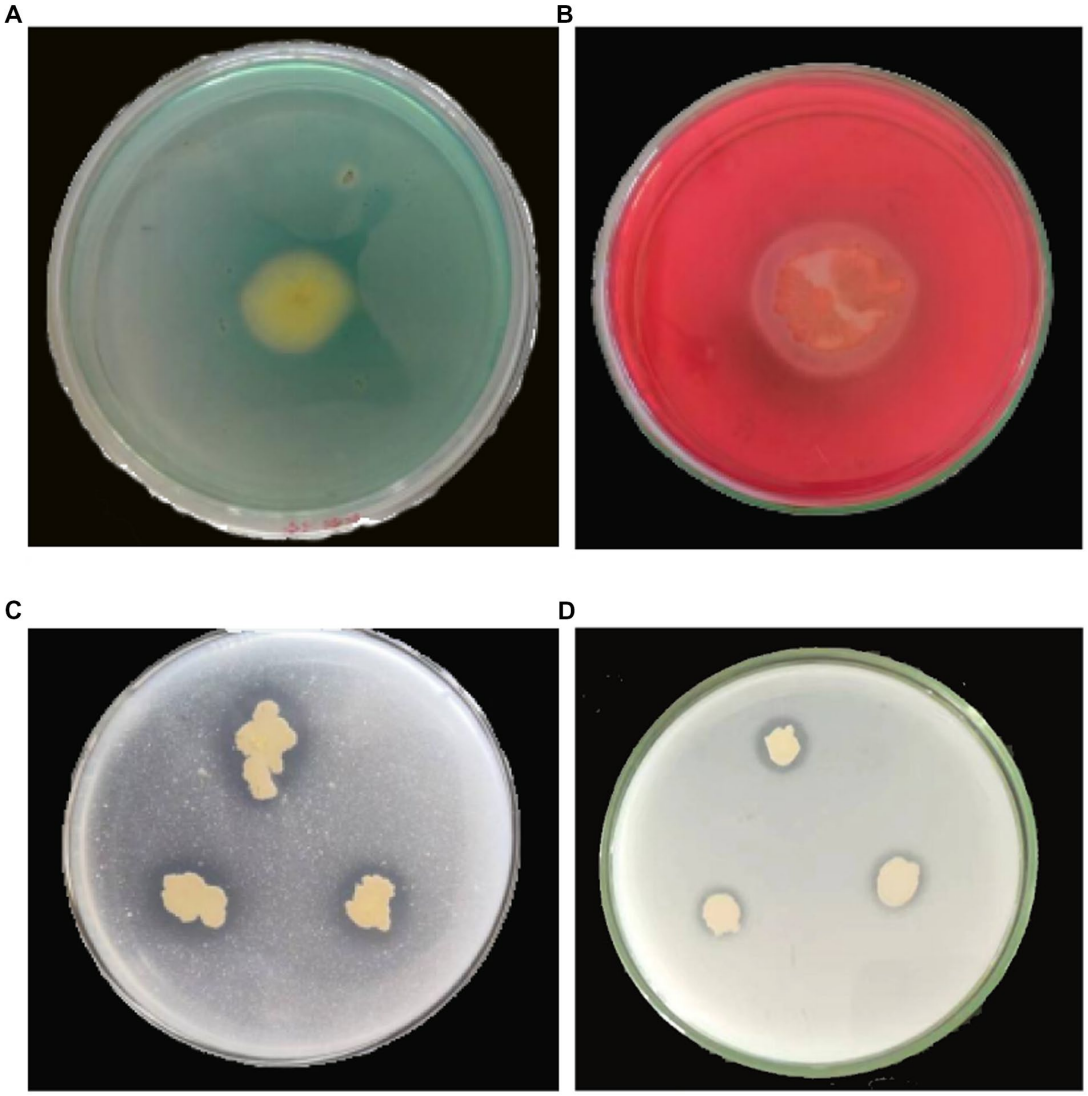
Comparison of *B. velezensis* YC89 genome sequences against five other Bacillus species genome sequences. **(A)** Synteny analysis of *B. velezensis* YC89, *B. velezensis* FZB42, *B. velezensis* CC09, *B. velezensis* SQR9, *B. velezensis* GS-1 and *B. amyloliquefaciens* DSM7 genomes. Pairwise alignments of the genomes were generated using MAUVE. The genome of strain YC89 was used as the reference genome. Boxes with the same color indicate syntenic regions. Boxes below the horizontal strain line indicate inverted regions. Rearrangements are shown and by colored lines. Scale is in nucleotides. **(B)** Venn diagram showing the number of clusters of orthologous genes shared and unique genes.

To evaluate the evolutionary distance among these sequenced strains in relation to several *Bacillus* strains, the genome sequence of YC89 was compared with that of four sequenced *B. velezensis* strains (GS-1, FZB42, CC09, and SQR9) and one *B. amyloliquefaciens* strain (DSM7). Alignments of *Bacillus* strains are presented in Figure 4A. Eight LCBs representing chromosomal similarity regions among the six strains were identified. Except for *B. velezensis* CC09, the collinearity level among the genomes of other strains was high, with only a few insertions, deletions, and inversions. The nucleotide similarity between *B. velezensis* YC89 and *B. velezensis* GS-1 was significantly higher than that between the other strains. Additionally, a pangenome analysis to compare YC89 with five other strains was performed. As presented in Figure 4B, 3,157 orthologous protein-coding genes constituted the core genome; 42 genes were unique to strain YC89. Annotation revealed that these specific genes encode a large number of transcriptional regulators, helicase domain proteins, hypothetical proteins, aminotransferases, transposases, drug resistance transporters, and chloramphenicol resistance proteins (Supplementary Table S3).

### 3.4. Analysis of CAZyme genes for *Bacillus velezensis* YC89 genome

**Figure 5:**
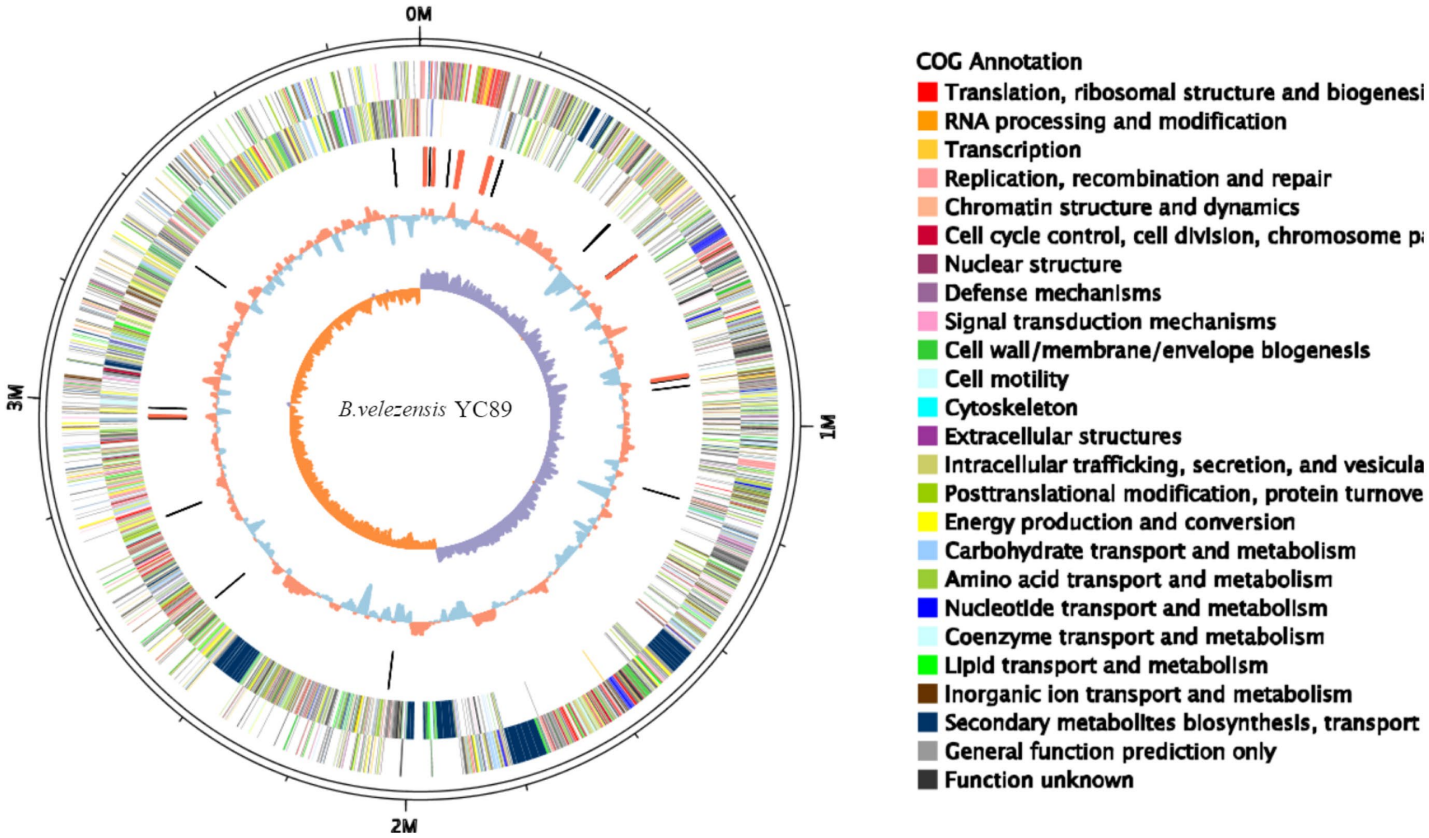
CAZymes gene classification in YC89 genome. GH, Glycoside Hydrolases; GT, Glycosyl Transferases, CE, Carbohydrate Esterases, AA, Auxiliary Activities, CBM, Carbohydrate-Binding Modules; PL, Polysaccharide Lyase.

CAZyme prediction of the *B. velezensis* YC89 strain revealed that 547 CAZyme genes; 5 had auxiliary activity, 87 were carbohydrate-binding modules (CBM), 46 carbohydrate esterases, 156 glycoside hydrolases, 250 glycosyl transferases, and three polysaccharide lyase genes (Figure 5). After a detailed classification of these six enzyme groups, 547 genes in the *B. velezensis* YC89 strain were assigned to 118 CAZyme families; the most abundant of these families was GT2 (108 members), which is responsible for transferring nucleotide bisphosphate sugars to substrates, such as polysaccharides and lipids. The CBM50 family contains 47 members that promote the antifungal activity of the enzyme by binding to the chitinous component of the fungal cell wall (Supplementary Table S4). Glucosidases (GH4 and GH13_29), chitinases (GH23 and GH18), and glucanases (GH5_2 and GH16) exhibited potential antifungal activities. These results suggested that CAZymes in YC89 played a significant role in antifungal activity.

### 3.5. Analysis of genes related to secondary metabolic and plant resistance induction

The YC89 genome was predicted using the online secondary metabolite gene cluster prediction tool antiSMASH; 12 secondary metabolite clusters were predicted, accounting for 18.62% of the whole genome (Table 2). These included two Transatpks gene clusters, two Transatpks-NRPS heterozygous gene clusters, two Terpene gene clusters, one NRPS gene cluster, one OtherKS gene cluster, one Lantipeptide gene cluster, one T3pks gene cluster, one Bacteriocin-NRPS gene cluster, and one Other gene cluster. Cluster 1 had 82% similarity to the surfactin synthesis gene cluster from BGC0000433; Cluster 2 had 7% similarity to the butirosin A/butirosin B synthesis gene cluster from BGC0000693; Cluster 5 had 7% similarity to the Macroprotein A/butirosin B synthesis gene cluster from BGC0000181; Cluster 6 had 100% similarity with the bacillaene synthetic gene cluster from BGC0001089; Cluster 7 had 100% similarity with the fengycin synthetic gene cluster from BGC0001095; Cluster 10 had 100% similarity with the bacillibactin synthetic gene cluster from BGC0000176; Cluster 11 had 100% similarity with the bacillibactin synthetic gene cluster from BGC0000309. Clusters 3, 4, 8, and 9 were functionally unknown, with no homologous sequences matched in the MIBiG accession database, suggesting that these secondary metabolic gene clusters may encode secondary metabolites that generate novel structures and warrant further isolation and identification.

**Table 2:** Putative gene clusters that encoded for secondary metabolites in YC89.

Gene clusters	Types	Most Similar Known Clusters	Size (bp)	MIBiG-ID (% of genes show similarity)	Bioactivity
Cluster 1	NRPS	Surfactin	75,559	BGC0000433 (82%)	Antibacterial
Cluster 2	PKS-like	Butirosin A/butirosin B	41,245	BGC0000693 (7%)	–
Cluster 3	Terpene	–	17,334		–
Cluster 4	Lantipeptide-class-ii	–	28,890		–
Cluster 5	transAT-PKS	Macrolactin H	87,814	BGC0000181 (100%)	Antibacterial
Cluster 6	transAT-PKS, T3PKS, transAT-PKS-like, NRPS	Bacillaene	100,786	BGC0001089 (100%)	Antibacterial and antifungal
Cluster 7	NRPS, transAT-PKS, betalactone	Fengycin	134,712	BGC0001095 (100%)	Antibacterial and antifungal
Cluster 8	Terpene	–	21,884		–
Cluster 9	T3PKS	–	41,101		–
Cluster 10	transAT-PKS	Difficidin	93,798	BGC0000176 (100%)	Antibacterial
Cluster 11	NRPS, RiPP-like	Bacillibactin	51,798	BGC0000309 (100%)	Iron-acquisition and antibacterial
Cluster 12	Other	Bacilysin	41,419	BGC0001184 (100%)	Antibacterial

Based on numerous reported examples of elicitors, genes encoding resistance inducers were selected for comparison among strains YC89, FZB42, and GS-1 using BLAST. As presented in Table 3, the genes encoding several elicitors, such as 2, 3-butanediol, acetoin, peptidoglycan, and EF-Tu, were detected in YC89, FZB42, and GS-1. However, the sequence identity between YC89 and GS-1 was higher than that between YC89 and GS-1. This finding indicates that although all three strains exhibited similar effects on inducing resistance, they may have certain differences in their efficiency to induce plant disease resistance owing to different gene similarities.

**Table 3:** Comparison of genes involved in synthesis of resistance inducers in strain YC89, FZB42, and GS-1.

Resistance inducers	Genes	Size (bp)	Plant resistance type	Product type	Identity (%)	
					FZB42	GS-1
2,3-butanediol	ilvN	519	ISR	Acetolactate synthase small subunit	98.65%	99.81%
2,3-butanediol	ilvB	1725	ISR	Acetolactate synthase 3 catalytic subunit	98.05%	99.32%
Acetoin	alsS	1713	ISR	Acetolactate synthase	99.12%	99.53%
Methanethio	metC	1,176	ISR	Cystathionine beta-lyase	97.28%	99.74%
Methanethio	mmuM	948	ISR	Homocysteine S-methyltransferase	97.68%	99.68%
Methanethio	metE	2,289	ISR	5-methyltetrahydropteroyltriglutamate–homocysteine S-methyltransferase	97.68%	99%
Isoprene	ispE	870	ISR	4-diphosphocytidyl-2-C-methyl-D-erythritol kinase	98.62%	99.54%
Isoprene	ispD	699	ISR	2-C-methyl-D-erythritol 4-phosphate	99%	99.86%
Isoprene	ispF	477	ISR	2-C-methyl-D-erythritol 2,4-cyclodiphosphate synthase	98.11%	100%
Isoprene	fni	1,050	ISR	Isopentenyl-diphosphate Delta-isomerase	97.43%	99.14%
Peptidoglycan	dacA	1,332	PTI	Carboxypeptidase	99.25%	99.62%
Flagellin	flgL	918	PTI	Flagellin	97.39%	99.89%
EF-Tu	tuf	1,191	PTI	Elongation factor Tu	99.58%	99.83%

### 3.6. Genes related to plant growth promotion in the *Bacillus velezensis* YC89 genome

Beneficial bacteria promote plant growth by influencing nutrient uptake, most genes related to plant growth promotion and protection were detected in *B. velezensis* YC89 (Table 4). IAA is an important phytohormone that controls cell enlargement and tissue differentiation in plants; eight genes related to IAA biosynthesis have been identified in strain YC89. Nitrogen, phosphorus, and potassium are essential for plant growth and development. One gene cluster, nar (H–K), nasD, and nirD, was predicted to be involved in nitrate transport and reduction. A gene cluster consisting of three genes, pst (ABC), was found in the YC89 genome and was predicted to be involved in phosphate solubilization. KtrC and yugO were found in the YC89 genome and predicted to be involved in potassium uptake. In addition, two genes, mgtE and corA, were predicted to be involved in Mg transport.

**Table 4:** Genes related to plant growth promotion in the *B. velezensis* YC89 genome.

ID	Gene	Gene product	Function
MJ920_11000	trpA	Tryptophan synthase subunit alpha	Indole-3-acetic acid biosynthesis
MJ920_11015	trpC	Indole-3-glycerol phosphate synthase	
MJ920_11020	trpD	Anthranilate phosphoribosyltransferase	
MJ920_11025	trpE	Anthranilate synthase component I	
MJ920_11030	aroH	Chorismate mutase	
MJ920_11035	aroB	3-dehydroquinate synthase	
MJ920_11040	aroC	Chorismate synthase	
MJ920_05905	trpS	Tryptophan–tRNA ligase	
MJ920_12270	pstC	Phosphate ABC transporter permease subunit	Phosphate solubilization
MJ920_12255	pstB	Phosphate ABC transporter ATP-binding protein PstB	
MJ920_12260	pstB	Phosphate ABC transporter ATP-binding protein PstB	
MJ920_12265	pstA	Phosphate ABC transporter permease	
MJ920_17855	narH	Nitrate reductase subunit beta	Nitrate transport and reduction
MJ920_17820	narI	Nitrate reductase subunit gamma	
MJ920_17825	narJ	Nitrate reductase molybdenum cofactor assembly chaperone	
MJ920_17830	narK	Nitrate transporter NarK	
MJ920_01690	nirD	Nitrite reductase small subunit NirD	
MJ920_01695	nasD	NADPH-nitrite reductase	
MJ920_07485	ktrC	Ktr system potassium transporter KtrC	Potassium transporter
MJ920_14795	yugO	Potassium channel family protein	
MJ920_06895	mgtE	Magnesium transporter	Magnesium utilization
MJ920_04005	corA	Magnesium/cobalt transporter CorA	

### 3.7. Determining the biological activity of *Bacillus velezensis* YC89 strains

**Figure 6:**
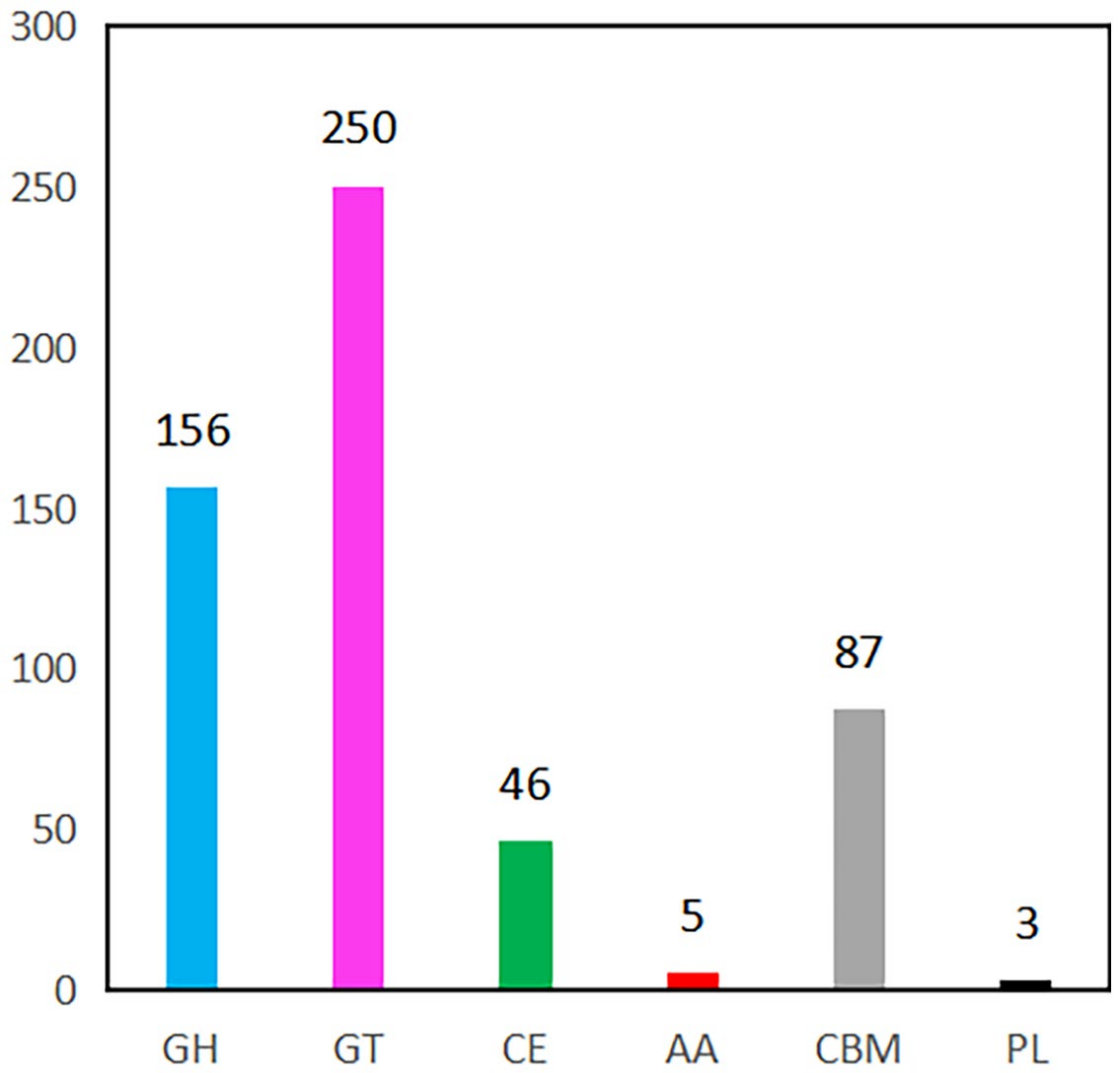
Determination of biological activity of *B. velezensis* YC89. **(A)**: siderophore, **(B)**: cellulase, **(C)**: Monkina, **(D)**: NBRIP.

To investigate the biocontrol mechanism of YC89, siderophore production, phosphate solubilization, and cellulase production of the YC89 strain were determined. The results revealed that the strain inoculated on the CAS plate produced a yellow transparent aperture; however, the strain inoculated on a solid phosphate solubilization medium with tricalcium phosphate and calcium phytate as substrate media produced transparent apertures, and a cellulose hydrolysis circle was formed in the carboxymethyl cellulase identification medium. This indicated that YC89 could produce siderophores, dissolve phosphorus, and produce cellulase (Figure 6).

### 3.8. Biocontrol activity of YC89 against *Colletotrichum falcatum*

#### 3.8.1. Analysis of plant defense-related enzymes

**Figure 7:**
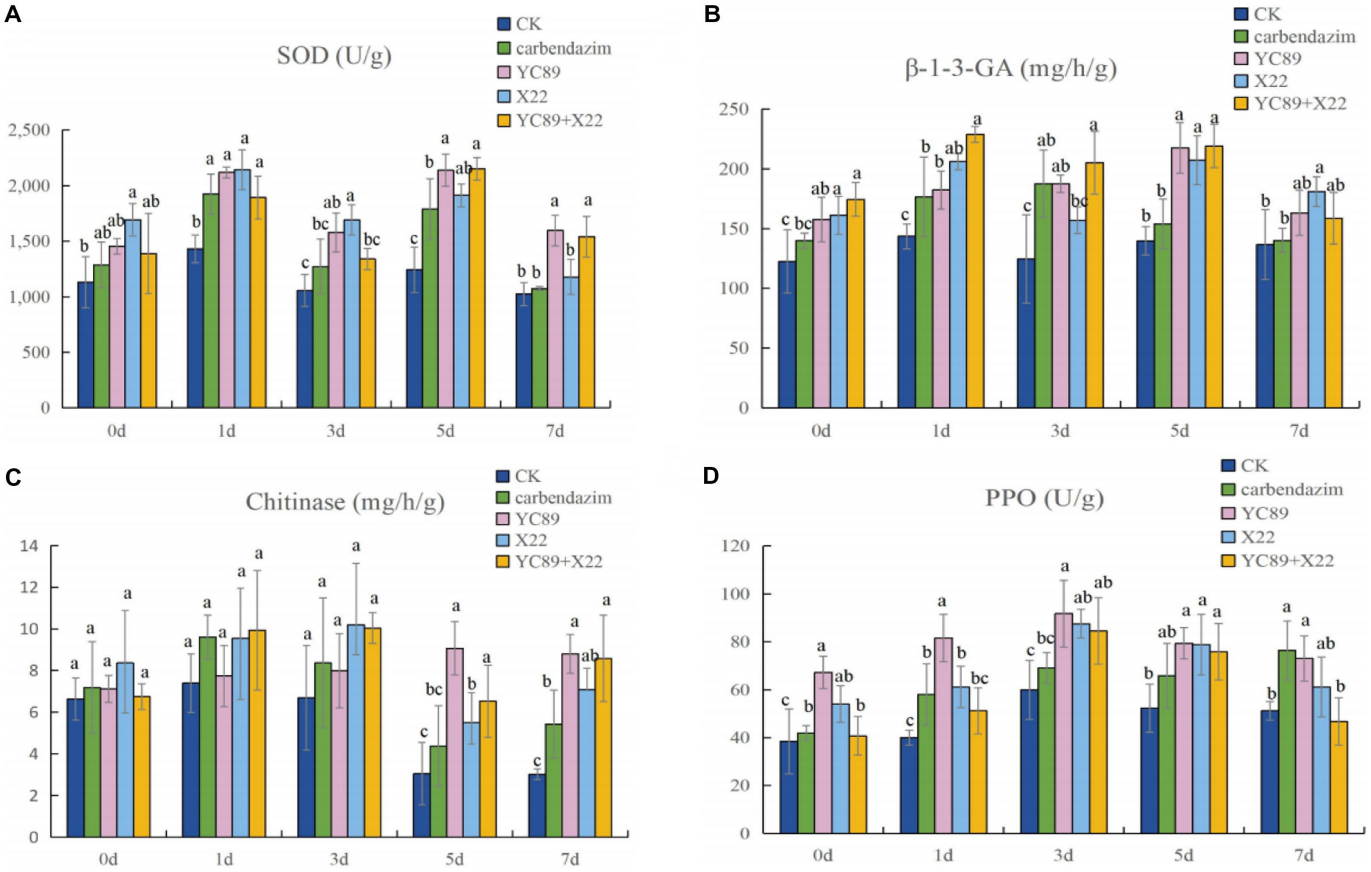
Plant Defense-Related Enzyme Analysis. **(A)** Superoxide dismutase activity (SOD), **(B)** β-1-3- glucase activity, **(C)** chitinase activity, **(D)** Polyphenol oxidase activity (PPO).

In order to determine the effect of biocontrol bacteria on the activity of disease resistance-related enzymes, *C. falcatum* was inoculated after inoculation of biocontrol bacteria, and the activities of SOD, β-1,3-GA, chitinase and PPO were measured at different times after inoculation of *C. falcatum*. The results are shown in Figure 7. Compared with the control treatment group, the biocontrol bacteria treatment group could increase the activities of SOD, β-1,3-GA, chitinase and PPO in sugarcane leaves and maximize their activities to cope with the invasion of *C. falcatum*. The SOD enzyme activity of sugarcane leaves reached the highest value 5 days after inoculation of *C. falcatum*, at this time, the enzyme activity difference was the largest. The enzyme activity of the biocontrol bacteria treatment group was significantly higher than the CK, in which the enzyme activity of YC89 treatment group was 41.90% higher than the CK, and 16.32% higher than the carbendazim treatment group (Figure 7A). The β-1,3-GA enzyme activity peaked on day 1 and 5 after inoculation of the *C. falcatum*, and the difference in enzyme activity between treatments was not significant on day 1, but was significantly higher than the CK. On the fifth day, the enzyme activity of biocontrol bacteria treatment group was significantly higher than the carbendazim treatment group and CK treatment group (Figure 7B). The effect of each treatment group on the chitinase activity was not significant from 0 to 3 days after inoculation with *C. falcatum*, and the difference between treatments was not significant. However, after the fifth day, the difference in chitinase activity was significant, with the YC89 treatment group and YC89 + X22 treatment group having significantly higher enzyme activity than the CK and carbendazim treatment groups (Figure 7C). PPO enzyme activity reached its peak three days after inoculation of *C. falcatum*, at this time, the enzyme activity difference was the largest, and the enzyme activity of YC89 treatment group was significantly higher than the CK and carbendazim treatment group (Figure 7D).

#### 3.8.2. Evaluation of *Bacillus velezensis* YC89 for biocontrol potential and growth promotion under greenhouse conditions

The pot-control effects of the biocontrol strains were also determined (Table 5). The results revealed that strains YC89 and X22 had certain control effects on sugarcane red rot and that the control effect was greater than that of carbendazim. The control effect of strain YC89 on sugarcane red rot disease was 61.91%. The control effect of the X22 strain on sugarcane red rot was worse than the YC89; however, the relative control effect of the mixed inoculation of the two strains was slightly different from that of YC89 alone. In addition, the plant growth-promoting capabilities of YC89 were evaluated using greenhouse experiments. As presented in Table 5, the growth parameters (plant height, stem diameter, and root length) of sugarcane subjected to the YC89 treatment were significantly higher than the control treatment. Collectively, the potted results indicated that the YC89 strain could control the red rot disease of sugarcane and promote the growth of sugarcane plants (Supplementary Figure S5).

**Table 5:** Evaluation of biocontrol efficacy of *B. velezensis* YC89 and its effect on plant growth parameters in sugarcane in greenhouse.

	Plant height (cm)	Stem diameter (mm)	Root length (cm)	Disease index	Biocontrol effect
CK	50.93 ± 2.55c	8.44 ± 0.24b	37.33 ± 3.16ab	66.22 ± 1.60a	
Carbendazim	51.61 ± 1.67c	8.10 ± 0.38b	33.04 ± 2.27b	57.47 ± 1.05b	13.21%
YC89	65.52 ± 2.74a	9.72 ± 0.48a	42.32 ± 2.60a	25.22 ± 1.55d	61.91%
X22	54.67 ± 1.80bc	8.42 ± 0.28b	35.63 ± 2.68ab	48.85 ± 1.09c	26.24%
YC89 + X22	59.93 ± 1.74ab	9.22 ± 0.45ab	37.77 ± 1.72ab	26.88 ± 1.83d	59.41%

CK, water treatment group.

## 4. Discussion

The genus *Bacillus* covers a large number of species with a high degree of similarity among many *Bacillus* species (in terms of the current classification), making it difficult to effectively determine the taxonomic status of strains using traditional classification methods (Hamdache et al., 2013). Several *B. amyloliquefaciens* are now reported as *B. velezensis*, such as type strain *B. amyloliquefaciens* FZB42 (Schilling et al., 2018), *B. amyloliquefaciens*vb7 (Saravanan et al., 2021), *B. amyloliquefaciens* SQR9 (Feng et al., 2018) so on. To determine the relationship between YC89 and *Bacillus* spp. strains, phylogenetic trees based on 16S rRNA gene sequences were constructed. These results indicate that YC89 is closely related to *B. velezensis* FZB42 and *B. amyloliquefaciens*. In order to be more accurate, we constructed a genome-wide phylogenomic tree, the phylogenetic analysis showed that the YC89 strain and *B. velezensis* GS-1 were in the same branch with the highest similarity.

Biological control, which has become an effective measure, is one of the safest and most environmentally friendly methods of plant disease control. Among biological control bacteria, *Bacillus* spp. are believed to be effective. The primary mechanisms used by *Bacillus* to control plant diseases include antagonism, competition, and induction of disease resistance in plants, however, they can also enhance their resistance by promoting plant growth, which is usually synergistic (Backer et al., 2018; Rodriguez et al., 2019). However, endophytic bacteria can promote the uptake and utilization of nutrients, such as nitrogen, phosphorus, and potassium, by host plants, promote plant growth and enhance their resistance, and produce certain plant growth hormones, such as IAA and indoleacetonitrile, which can directly promote plant growth (Pii et al., 2015). Pot experiments using YC89 to treat sugarcane plants revealed an increase in shoot height and root length. In this study, the YC89 genome annotation demonstrated that a large number of YC89 genes are involved in plant growth by enhancing nutrient uptake and availability. In the YC89 genome, the nitrate transporter nark and the nitrate reductases narH-narI-narJ, nirD, and nasD were found. These gene clusters were predicted to be involved in nitrate transport and reduction (Wray et al., 1994). Additionally, genes involved in potassium transport were identified in the YC89 genome; two genes, *ktrC* and *yugO*, that have been identified and characterized as potassium transporters in *Bacillus* spp., were identified in the YC89 genome. In a recent full-genome analysis of *B. velezensis* FZB42, *mgtE*, and *corA* were predicted to be involved in Mg uptake and detoxification of heavy metal ions in host plants (Schilling et al., 2018). Hence, *MgtE* and *corA* likely have similar roles in YC89. Biocontrol bacteria can compete with pathogens for iron to inhibit pathogen growth; in this study, strain YC89 was able to produce siderophores. In addition, a cluster (bacillibactin) was found in the YC89 genome that predicted putative iron availability.

In recent years, research has focused on investigating the ability of *B. velezensis* to produce various secondary metabolites and enzymes with broad-spectrum resistance to plant pathogens (Wang et al., 2021). Antimicrobial cyclic lipopeptides synthesized by the NRPS and polyketides synthesized by the polyketide synthesis pathway form the basis of their broad-spectrum antibacterial activity. Cyclic lipopeptides primarily include the three families of surfactin, iturin, and fengycin, all of which can induce systemic resistance to enhance plant defense against pathogens (Jourdan et al., 2009; Wu et al., 2018). However, the gene cluster encoding iturin was not found in the YC89 genome. Polyketides, including macrolactin, bacillaene, and diffidin, can be used as antibiotics, antifungals, or natural insecticides. The main polyketides identified in *Bacillus* are derived from *B. subtilis* and *B. velezensis*, among which *B. velezensis* is particularly abundant in polyketides (Baek, 2020). Using whole genome sequencing, Zhang et al. (2022) found that *B. velezensis* GS-1 was able to produce the lipopeptide compounds, surfactin, fengycin, and plantazolicin, and confirmed the inhibitory effect of lipopeptide compounds on *M. oryzae*. Meanwhile, eight secondary metabolite-related gene clusters were identified in the *Bacillus velezensis* VB7 genome, the *Bacillus velezensis* VB7 was a potential antagonist for managing carnation infection by *Sclerotinia sclerotiorum*, cotton infection by tobacco streak virus, and groundnut bud necrosis infection of tomato (Saravanan et al., 2021). In the current study, the YC89 genome was predicted using the online secondary metabolite gene cluster prediction tool antiSMASH, which predicted 12 gene clusters, including four of unknown function and seven antibiotic synthesis gene clusters with high similarity (surfactin, macrolactin H, bacillaene, fengycin, difficidin, bacillibactin, and bacilysin). One antibiotic gene cluster (butirosin) showed only 7% similarity. Butirosin is an aminoglycoside antibiotic that is particularly effective against gram-negative bacteria (Kudo and Eguchi, 2009). Although 12 secondary metabolite gene clusters were identified by whole-genome sequencing, the isolation and extraction of secondary metabolites and their inhibitory effects requires further investigation.

The main component of the cell wall in most pathogenic fungi is chitin. Chitinase catalyzes chitin hydrolysis to produce N-acetylglucosamine, which destroys the structural integrity of the fungal cell wall (Zhang et al., 2018). Endophytic bacteria can also produce hydrolases, such as chitinase, cellulase, and glucanase. These hydrolases can destroy fungal cell structure and dissolve the cell wall, thereby inhibiting fungal growth (Verma and Gange, 2014). Indeed, plant cell walls are composed of a complex network of carbohydrates, such as cellulose, hemicellulose, pectin, proteins, and glycoproteins. CAZymes cleave polysaccharides and other structural compounds associated with similar microorganisms into oligomers and simple monomers for nutrient uptake by microorganisms (Stafford and Satur, 2017). CAZyme analysis showed that the *B. velezensis* YC89 genome contained 547 CAZyme genes, including those associated with cell wall degrading enzymes, such as β-1,3-glucanase, and chitinase; it was hypothesized that degradation of the cell wall of pathogenic fungi serves as an inhibitory mechanism of *B. velezensis* YC89. Thus, the CAZymes in *B. velezensis* YC89 strain likely have an important role in spatial competition and nutrient acquisition and play an integral part in suppressing the virulence of the sugarcane red rot pathogen.

Additionally, endophytes can induce systemic resistance in plants to resist pathogen infestation and reduce disease incidence. Plant-induced resistance is synthesized by a combination of physiological and biochemical factors, such as defense enzyme activity, lignin, disease-course-associated proteins, and multi-post compounds. SOD, PPO, gibberellinase, and β-1,3 glucanase (β-1,3-GA) resistance enzymes in plants play an important role in the defense against foreign pathogens, and their enzyme activity level is often used to indicate the strength of disease resistance in plants (Song et al., 2006). To understand the mechanism of *B. velezensis* F21-mediated ISR in *Fusarium* wilt, Jiang et al. (2019) determined the expression levels of defense-related genes and the activities of defense-related enzymes (CAT, POD, and SOD) in the roots of watermelon inoculated with *Fusarium oxysporum f.* sp. *niveum* (Fon) only and in those pretreated with *B. velezensis* F21 before inoculation with Fon. The results indicated that compared with the control treatment, *B. velezensis* F21 could increase the activities and prepare the defense-related enzymes for maximum activity in advance of Fon invasion. In this study, we measured the activity and relative efficacy of sugarcane leaf resistance enzymes in a pot test and identified that YC89 and X22 in the biocontrol solution increased SOD, PPO, gibberellinase, and β-1,3-GA activities. In fact, the relative efficacy of the treatment groups with a biocontrol solution was higher than that of the water and carbendazim groups. In addition, ten genes related to ISR were identified in *B. velezensis* YC89. These results demonstrate that *B. velezensis* YC89 induces similar systemic resistance in plants.

Although the optimal method for controlling sugarcane red rot includes the application of chemical fungicides, long-term use can lead to the development of resistance by the pathogenic bacteria, resulting in a decrease in treatment efficacy. Moreover, the frequent use of high-concentration chemical fungicides can cause the formation of pesticide residues, which pollute the environment and pose a severe threat to human health. As an alternative, various biocontrol agents have been implemented. In particular, *Pseudomonas* spp., *Trichoderma* spp., *Bacillus* spp., and *Streptomyces* spp. are the most effective biocontrol agents against sugarcane red rot (Wu et al., 2015). In this study, the relative efficacy of *B. velezensis* YC89 against sugarcane red rot was determined using a pot test, and the results showed that *B. velezensis* YC89 had a relative efficacy of 61.91% against sugarcane red rot, in addition to promoting the growth of sugarcane plants. Although the greenhouse pot experiment simulates the field environment to a large extent, compared to the field environment, greenhouse pots have controlled characteristics and less interference than plants in the field, so that the effects of the strain on the plant would be more pronounced in the greenhouse pots. The induction of biocontrol agents is easily affected by various factors in the field, including environment, temperature, climate, and terrain; therefore, further research is required in field trials of strain YC89.

The colonization of a single biocontrol bacterium in the soil is unknown and has a limited effect on the control of soil pathogens, thus affecting the promotion of their use. However, the synergistic effects among different biocontrol agents and strains are difficult to predict and may reduce the control effect (Xu et al., 2011; Pertot et al., 2017). In this study, strain YC89 was mixed with strain X22 to control sugarcane red rot; although the effect did not differ significantly from that of YC89 alone, it was superior to that of X22 alone, indicating that the biological control ability of strain YC89 was not inhibited by strain X22 after combined inoculation. Hence, given that many biocontrol agents are being used to control plant diseases, the effects of combining *B. velezensis* YC89 with other beneficial microorganisms warrants further investigation.

## 5. Conclusion

Our study findings indicate that *B. velezensis* YC89 could serve as a potential biocontrol agent for sugarcane red rot and promote sugarcane plant growth. Whole-genome and phylogenetic analyses revealed that YC89 belongs to *B. velezensis* and is closely related to *B. velezensis* GS-1. Genome analysis demonstrated that YC89 harbors multiple genes related to IAA production, nitrogen fixation, phosphate solubilization, antifungal activity, and resistance inducer biosynthesis. The underlying biocontrol mechanisms can be inferred as follows: (1) production of active antibacterial substances, such as antibiotics and hydrolases; (2) significant promotion of plant growth through nitrogen fixation, phytohormone synthesis, and phosphorus augmentation; and (3) induction of ISR in plants for defense against pathogenic bacterial infection. Overall, the features of *B. velezensis* YC89 make it a potential biocontrol agent and biofertilizer. These results will contribute to in-depth research on sugarcane plant growth promotion and biocontrol mechanisms.

## Data availability statement

The datasets presented in this study can be found in online repositories. The names of the repository/repositories and accession number(s) can be found in the article/supplementary material.

## Author contributions

FL, LH, and LX designed the experiments. LX conducted most of the experiments. LL, YL, and XR performed a small number of experiments and provided experimental methods. YD, HL, ZQ, and QS carried out the statistical analyses and organized the data. LX wrote the manuscript. FL and LH revised the manuscript. All authors contributed to the article and approved the submitted version.

## Funding

This project was supported by the Major Science and Technology Special Project of Yunnan Province (202202AE090021), Yunnan Provincial Key Laboratory of Crop Production and Smart Agriculture Special Project (202105AG070007), Yunnan Provincial Education Department Scientific Research Fund Project (2022 J0286) and Yunnan Province Modern Agricultural Sugarcane Industry Technical System Construction Project (2022–2023).

## Conflict of interest

The authors declare that the research was conducted in the absence of any commercial or financial relationships that could be construed as potential conflict of interest.

## Publisher’s note

All claims expressed in this article are solely those of the authors and do not necessarily represent those of their affiliated organizations, or those of the publisher, the editors and the reviewers. Any product that may be evaluated in this article, or claim that may be made by its manufacturer, is not guaranteed or endorsed by the publisher.

## Abbreviations

β-1,3-GA, β-l,3-glucanase
POD: peroxidase
PPO: polyphenol oxidase
SOD: superoxide dismutase
ISR: induced systemic resistance
CDS: coding sequences
NCBI: National Center for Biotechnology Information
LCBs: locally collinear blocks
ANI: average nucleotide identity
PDA: potato dextrose agar
NR: non-redundant
KEGG: Kyoto Encyclopedia of Genes and Genomes
GO: gene ontology
COG: Cluster of Orthologous Groups
CAZyme: carbohydrate-active enzyme
BLAST: basic local alignment search tool
IAA: indole acetic acid
CAS: Chrome Azurol S
NRPS: non-ribosomal peptide synthetase
CK: water treatment group
