# Corrigendum: Complete genome sequence of biocontrol strain *Bacillus velezensis* YC89 and its biocontrol potential against sugarcane red rot

**DOI:** 10.3389/fmicb.2023.1235695

**Published:** 2023-07-11

**Authors:** Linyan Xie, Lufeng Liu, Yanju Luo, Xibing Rao, Yining Di, Han Liu, Zhenfeng Qian, Qingqing Shen, Lilian He, Fusheng Li

**Affiliations:** ^1^College of Agronomy and Biotechnology, Yunnan Agricultural University, Kunming, China; ^2^College of Resources and Environment, Yunnan Agricultural University, Kunming, China; ^3^Sugarcane Research Institute, Yunnan Agricultural University, Kunming, China

**Keywords:** *Bacillus velezensis*, biocontrol, sugarcane red rot, genome sequencing, biocontrol mechanism

In the published article, there was an error regarding the affiliations for Lufeng Liu. As well as having affiliation 2, they should also have “^1^ College of Agronomy and Biotechnology, Yunnan Agricultural University, Kunming, China” and “^3^ Sugarcane Research Institute, Yunnan Agricultural University, Kunming, China.”

In the published article, there was an error regarding the affiliations for Yining Di. As well as having affiliations 2 and 3, they should also have “^1^ College of Agronomy and Biotechnology, Yunnan Agricultural University, Kunming, China.”

In the published article, there was an error regarding the affiliations for Lilian He. As well as having affiliation 1, they should also have “^3^ Sugarcane Research Institute, Yunnan Agricultural University, Kunming, China.”

In the published article, there was an error regarding the affiliations for Fusheng Li. As well as having affiliation 1, they should also have “^3^ Sugarcane Research Institute, Yunnan Agricultural University, Kunming, China.”

A correction has been made to **Results**, “3.1. Genomic features and annotation of *Bacillus velezensis* YC89”, paragraph two. The sentence previously stated:

“Among these pathways, the most represented pathways included carbohydrate metabolism (242 genes, 6.52% of all CDS), followed by amino acid metabolism, and metabolism of cofactors and vitamin pathways (**Supplementary Figure S3**).”

The corrected sentence appears below:

“Among these pathways, the most represented pathways included carbohydrate metabolism (242 genes, 6.52% of all CDS), followed by amino acid metabolism, and metabolism of cofactors and vitamin pathways (**Supplementary Figure S2**).”

A correction has been made to **Results**, “3.2. Identification of strain YC89”, paragraph one. The sentence previously stated:

“These results indicate that YC89 is closely related to *B. velezensis* FZB42 and *B. amyloliquefaciens* 6B (**Supplementary Figure S3**). For an in-depth analysis of the taxonomic status of YC89 strain, a genomewide phylogenomic tree was constructed. The results revealed that strain YC89 formed a close genetic relationship with strain *B. velezensis* GS-1 (**Supplementary Figure S4**).”

The corrected sentence appears below:

“These results indicate that YC89 is closely related to *B. velezensis* FZB42 and *B. amyloliquefaciens* 6B (**Supplementary Figure S3A**). For an in-depth analysis of the taxonomic status of YC89 strain, a genome-wide phylogenomic tree was constructed. The results revealed that strain YC89 formed a close genetic relationship with strain *B. velezensis* GS-1 (**Supplementary Figure S3B**).”

A correction has been made to **Results**, “3.8.2. Evaluation of *Bacillus velezensis* YC89 for biocontrol potential and growth promotion under greenhouse conditions”, paragraph one. The sentence previously stated:

“Collectively, the potted results indicated that the YC89 strain could control the red rot disease of sugarcane and promote the growth of sugarcane plants (**Supplementary Figure S5**).”

The corrected sentence appears below:

“Collectively, the potted results indicated that the YC89 strain could control the red rot disease of sugarcane and promote the growth of sugarcane plants (**Supplementary Figure S4**).”

In the published article, there were errors in Figures 1–6 as published. The order of images was incorrect, such that the captions appeared alongside the wrong images. The corrected Figures 1–6 and their captions appear below.

**Figure 1:**
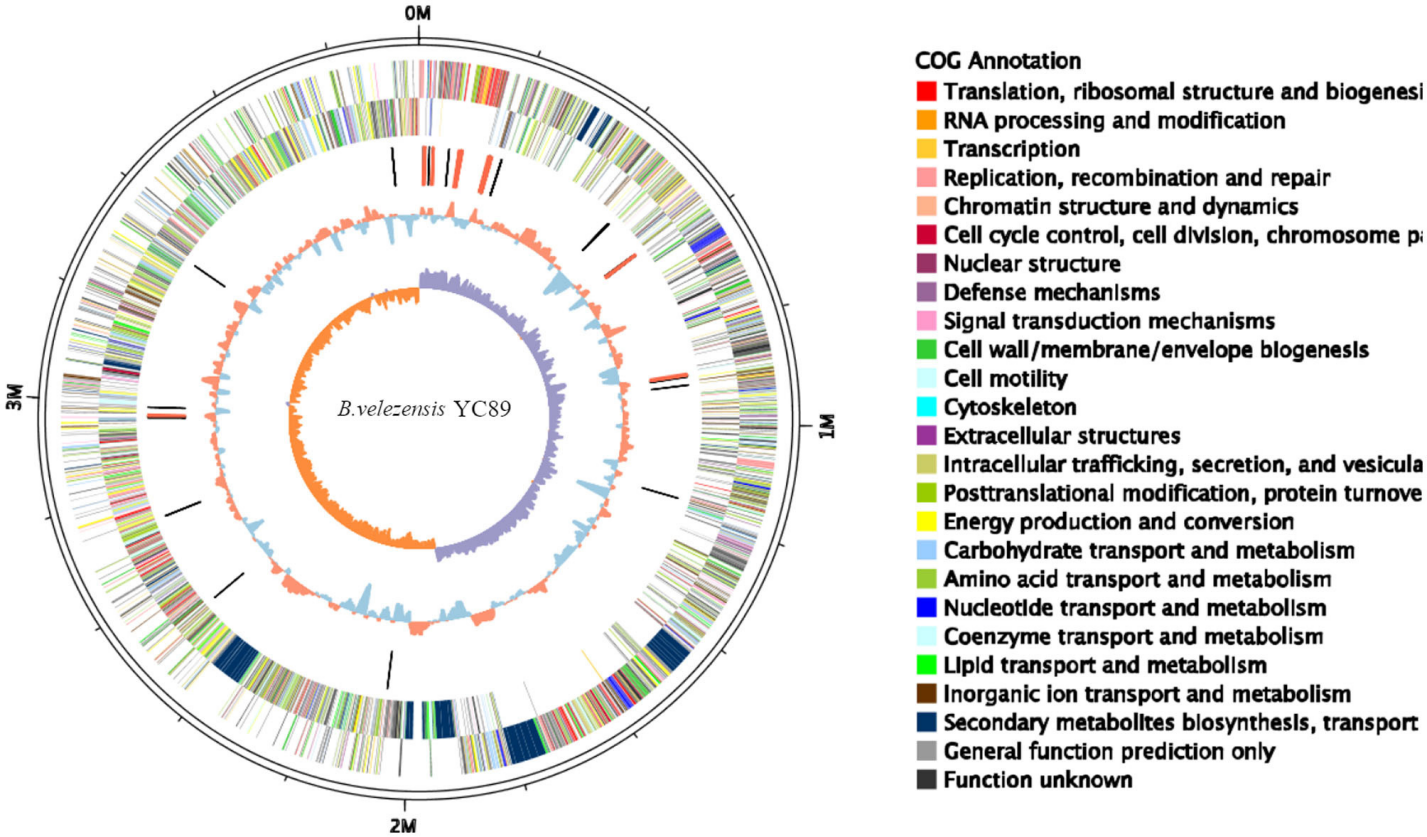
Circular genome map of *Bacillus velezensis* YC89. From the innermost to the outermost: ring 1 GC skew positive (Orange) and negative (purple); ring 2 for GC content, Orange is greater than the average, blue is less than the average; ring 3 for distribution of rRNAs (red) and tRNAs (black); ring 4 COG classifications of protein-coding genes on the reverse strand; ring 5 COG classifications of protein-coding genes on the forward strand; ring 6 for genome size (black line).

**Figure 2:**
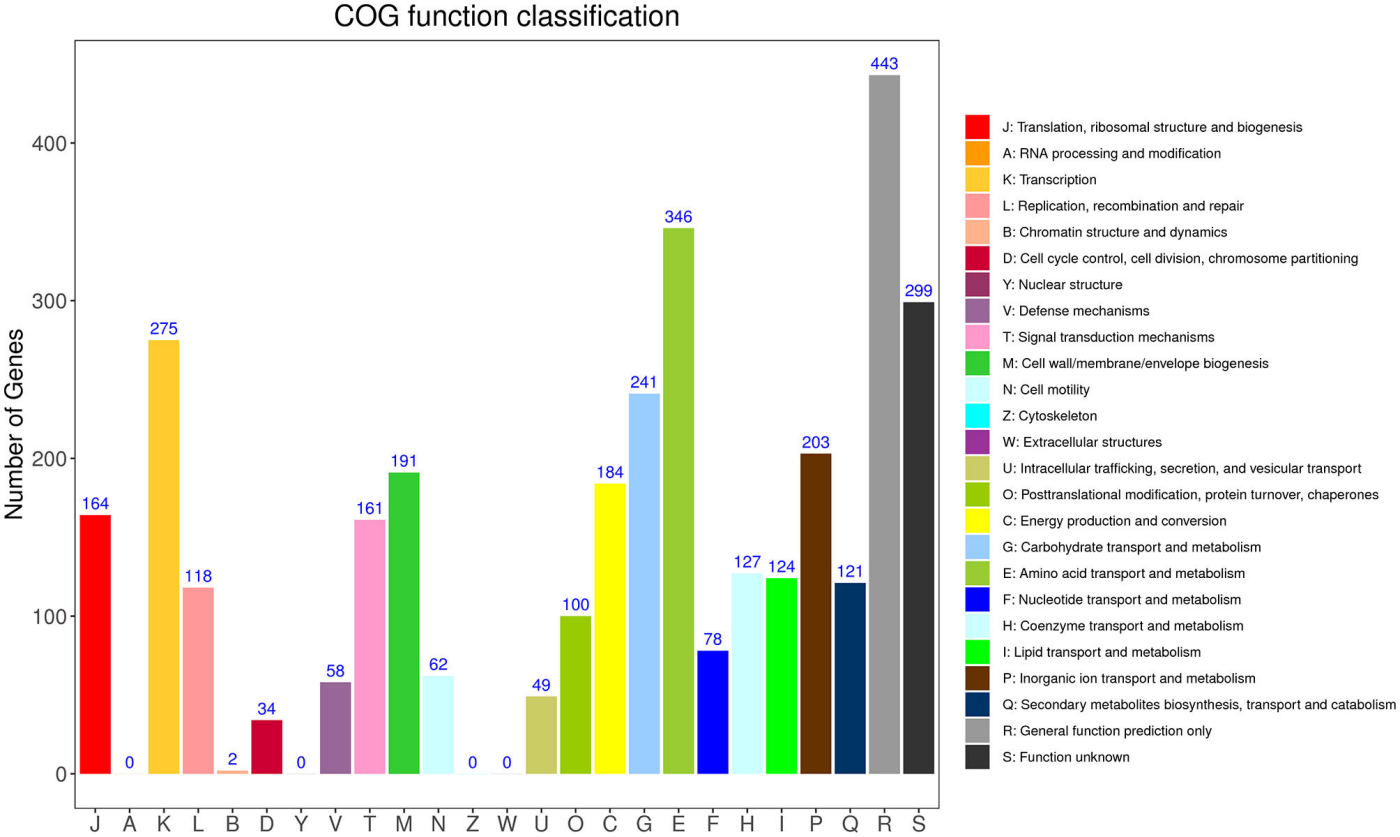
Distribution of genes across COG functional categories in the chromosome of YC89.

**Figure 3:**
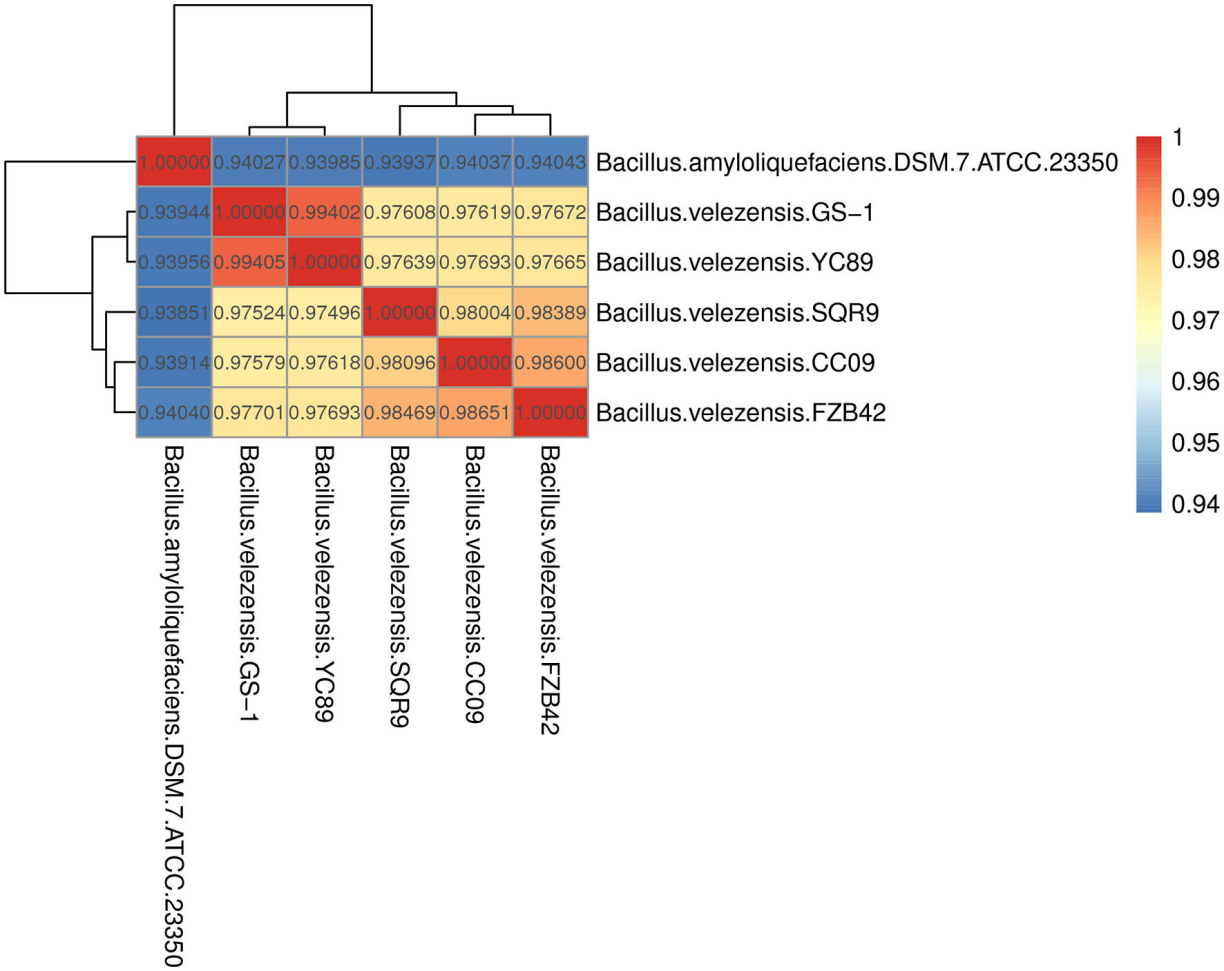
Heatmap of pairwise average nucleotide identity (ANI) values for whole genomes of YC89 and five other *Bacillus* species.

**Figure 4:**
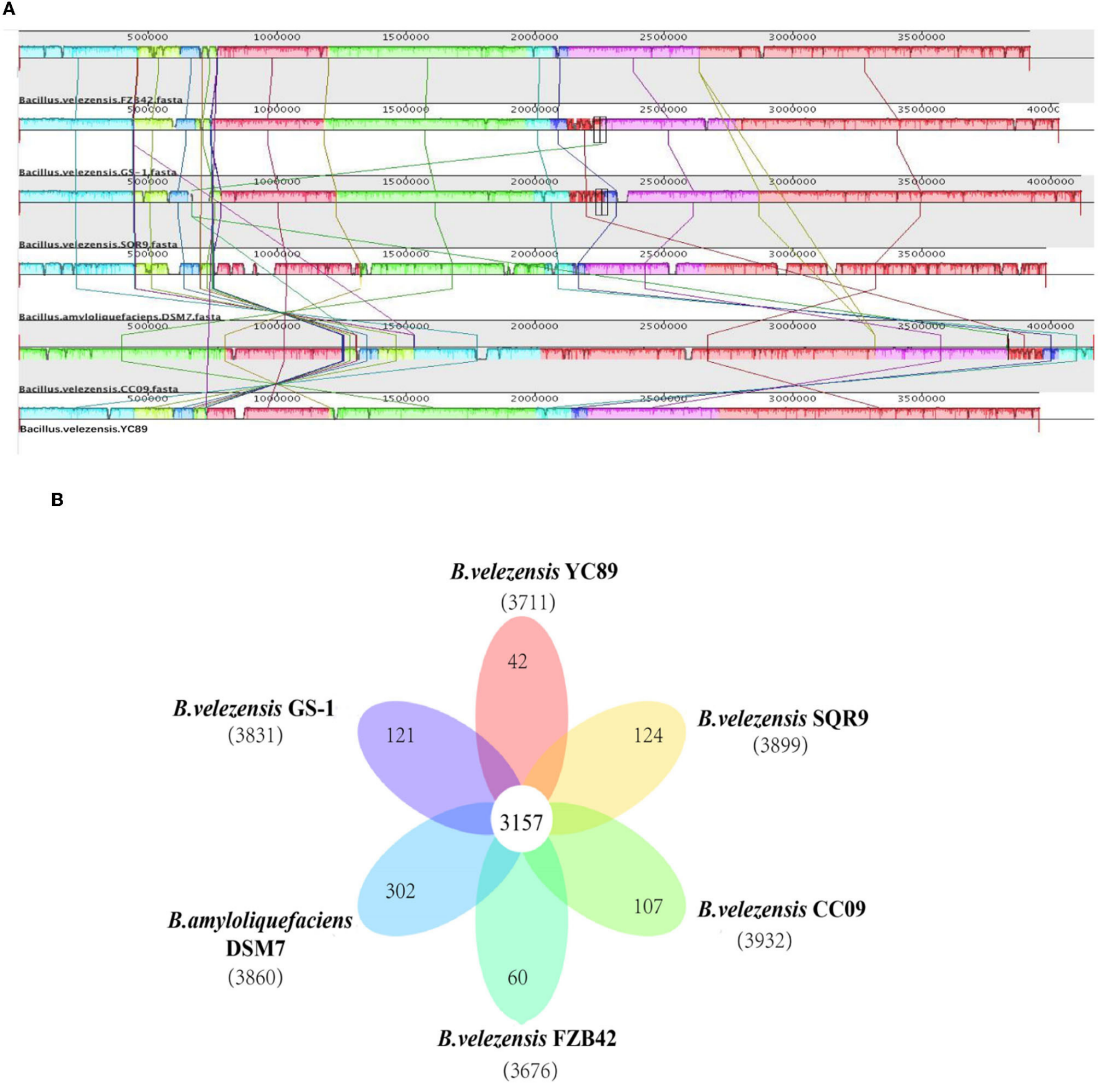
Comparison of *B. velezensi*s YC89 genome sequences against five other *Bacillus* species genome sequences. **(A)** Synteny analysis of *B. velezensis* YC89, *B. velezensis* FZB42, *B. velezensis* CC09, *B. velezensis* SQR9, *B. velezensis* GS-1 and *B. amyloliquefaciens* DSM7 genomes. Pairwise alignments of the genomes were generated using MAUVE. The genome of strain YC89 was used as the reference genome. Boxes with the same color indicate syntenic regions. Boxes below the horizontal strain line indicate inverted regions. Rearrangements are shown and by colored lines. Scale is in nucleotides. **(B)** Venn diagram showing the number of clusters of orthologous genes shared and unique genes.

**Figure 5:**
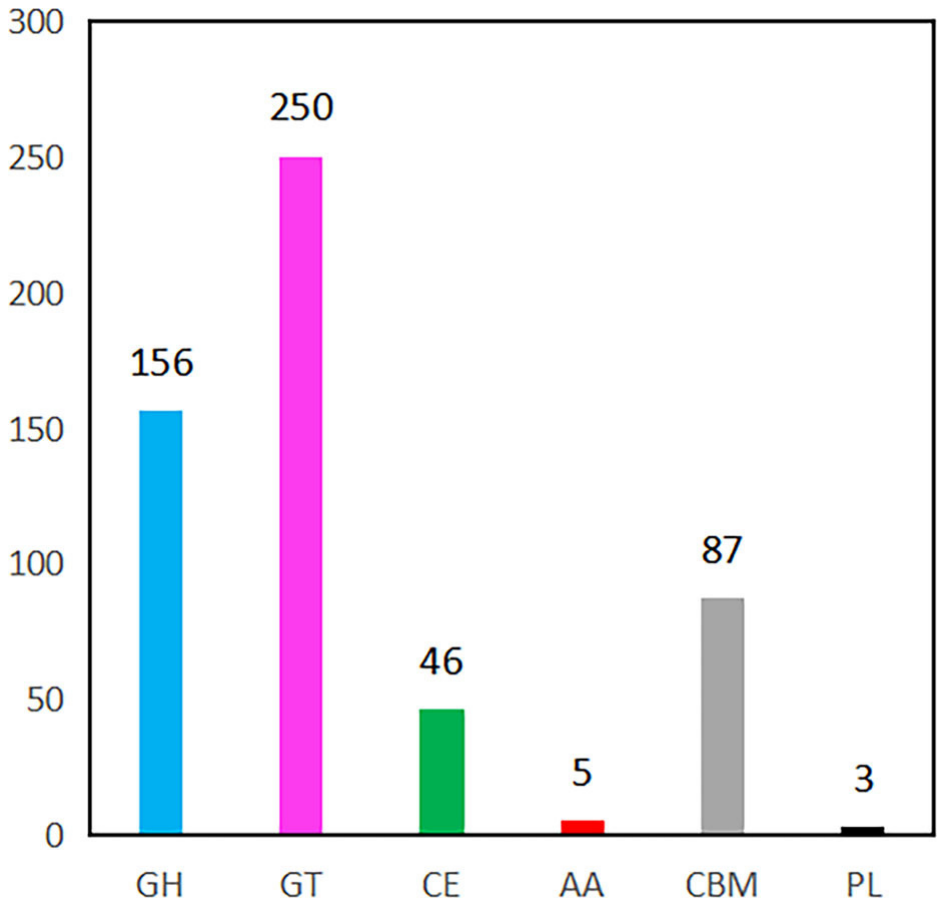
CAZymes gene classification in YC89 genome. GH, Glycoside Hydrolases; GT, Glycosyl Transferases, CE, Carbohydrate Esterases, AA, Auxiliary Activities, CBM, Carbohydrate-Binding Modules; PL, Polysaccharide Lyase.

**Figure 6:**
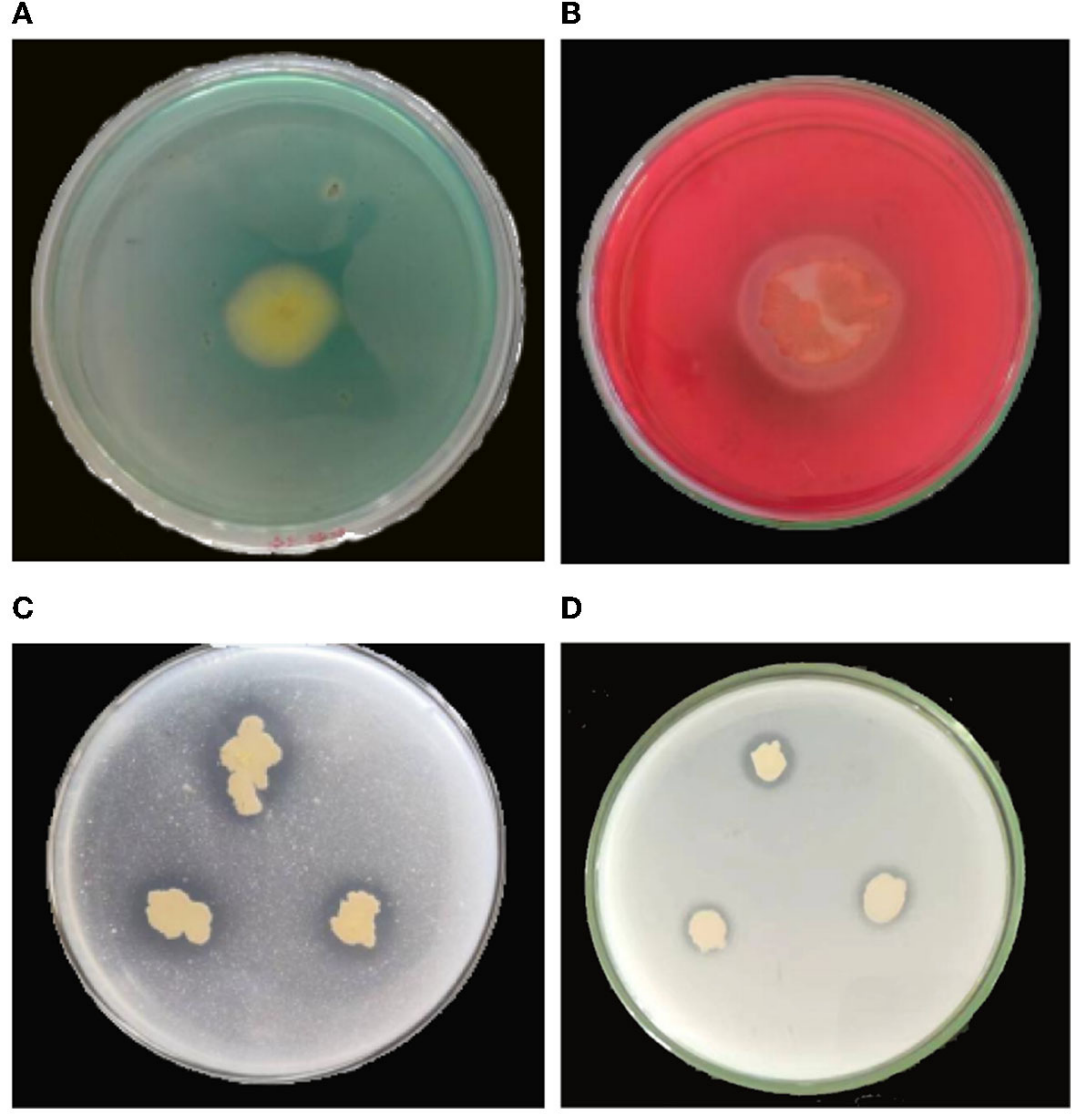
Determination of biological activity of *B. velezensis* YC89. **(A)**: siderophore, **(B)**: cellulase, **(C)**: Monkina, **(D)**: NBRIP.

The authors apologize for these errors and state that they do not change the scientific conclusions of the article in any way. The original article has been updated.

