# Supplementary material for: Complete genome sequence of biocontrol strain Bacillus velezensis YC89 and its biocontrol potential against sugarcane red rot
Source: Front Microbiol. 2023 Jun 2;14:1180474. doi: 10.3389/fmicb.2023.1180474 (PMC10275611; doi:10.3389/fmicb.2023.1180474)
Supplement: Supplementary file 1 [file Table_1.docx]

Supplementary Material

Complete genome sequence of biocontrol strain *B**acillus velezensis* YC89 and its biocontrol potential against sugarcane red rot

LYX, LLH*****, FSL*****

*** Correspondence:** Corresponding Author: email@uni.edu

# Supplementary Figures and Tables

For more information on Supplementary Material and for details on the different file types accepted, please see [here](https://www.frontiersin.org/guidelines/author-guidelines#supplementary-material).

## Supplementary Figures

##
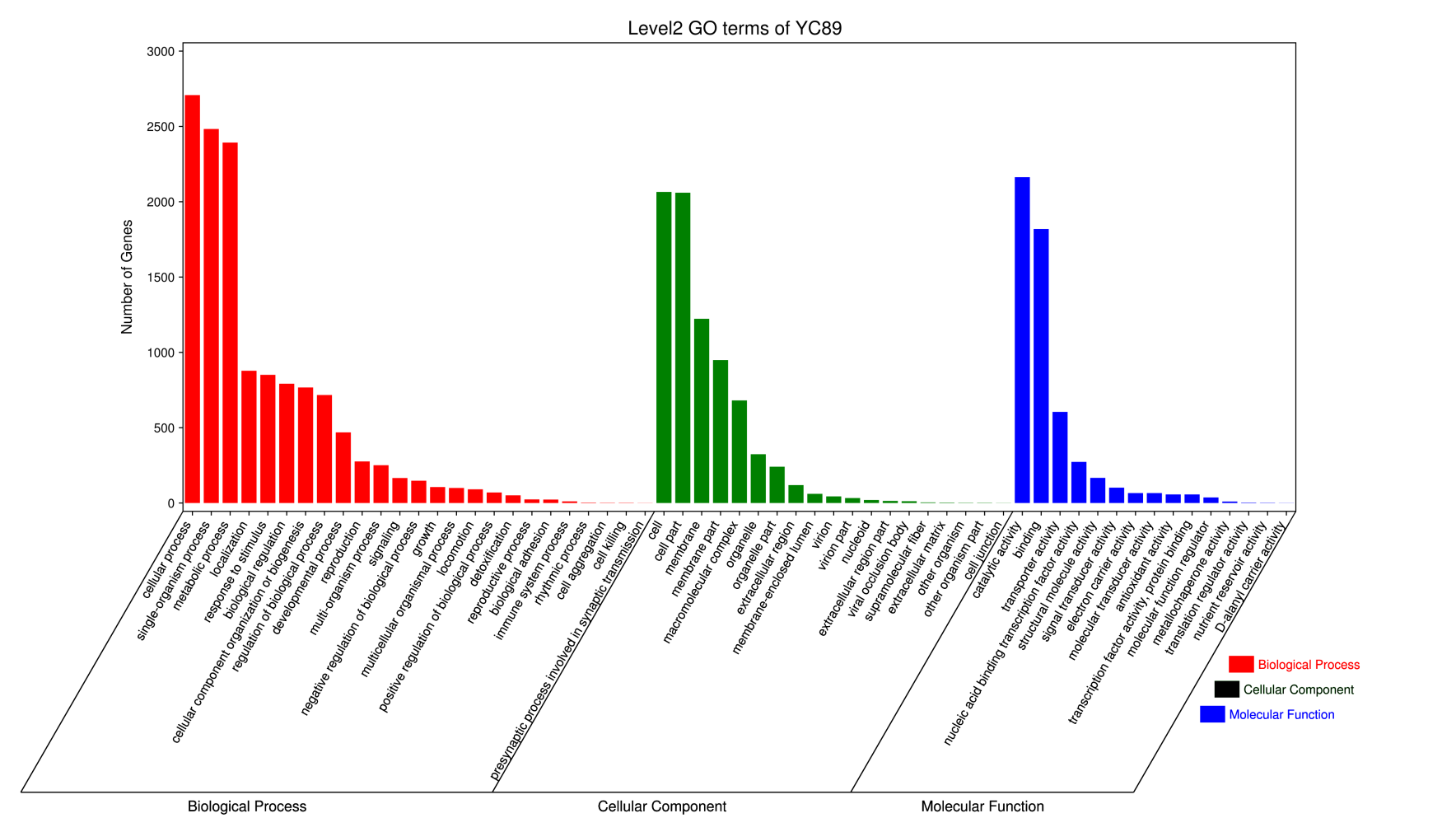


## **Supplementary Figure S1.** GO temre of YC89


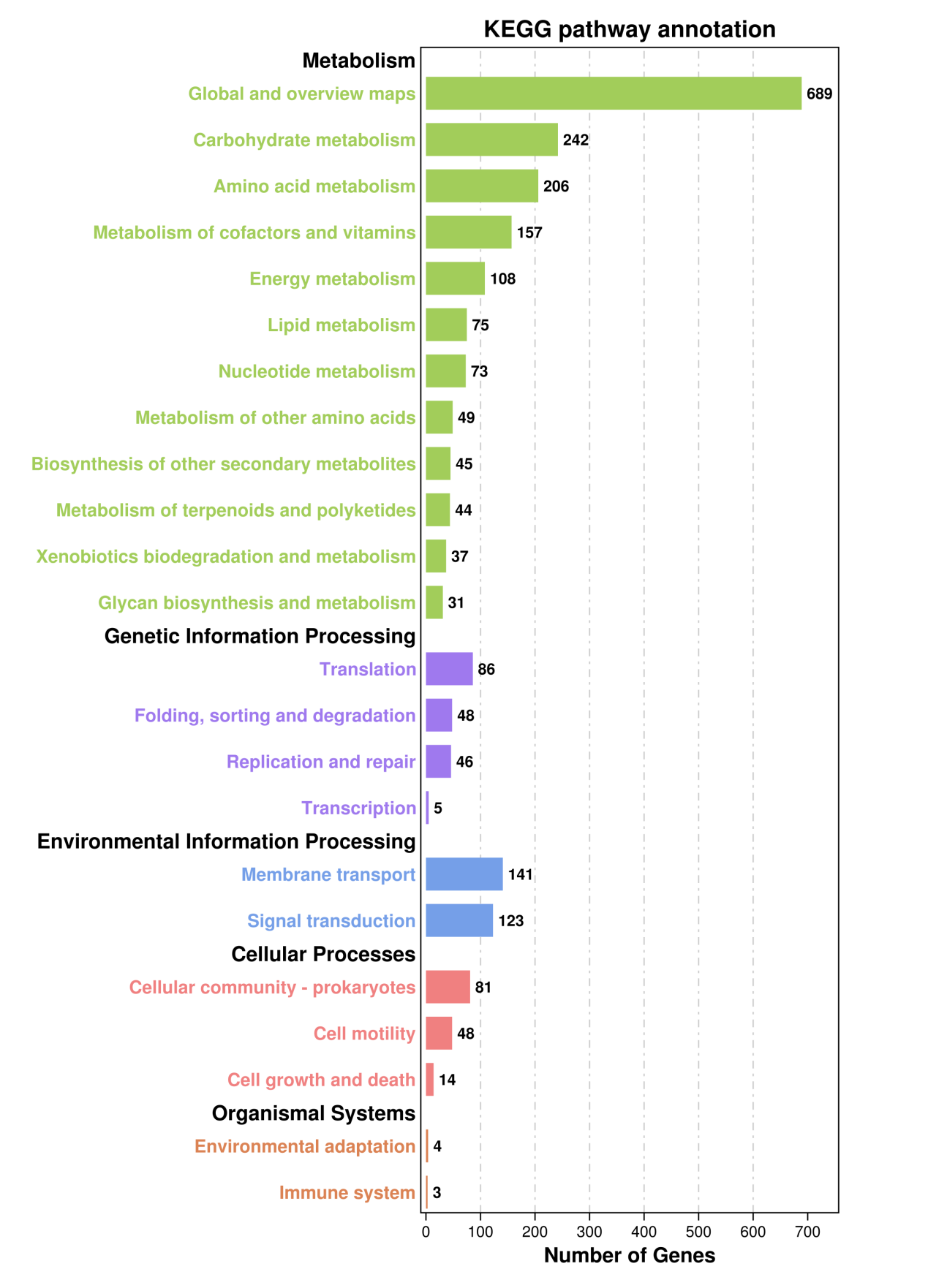


**Supplementary Figure S2:** Kyoto Encyclopedia of Genes and Genomes (KEGG) Pathway annotation


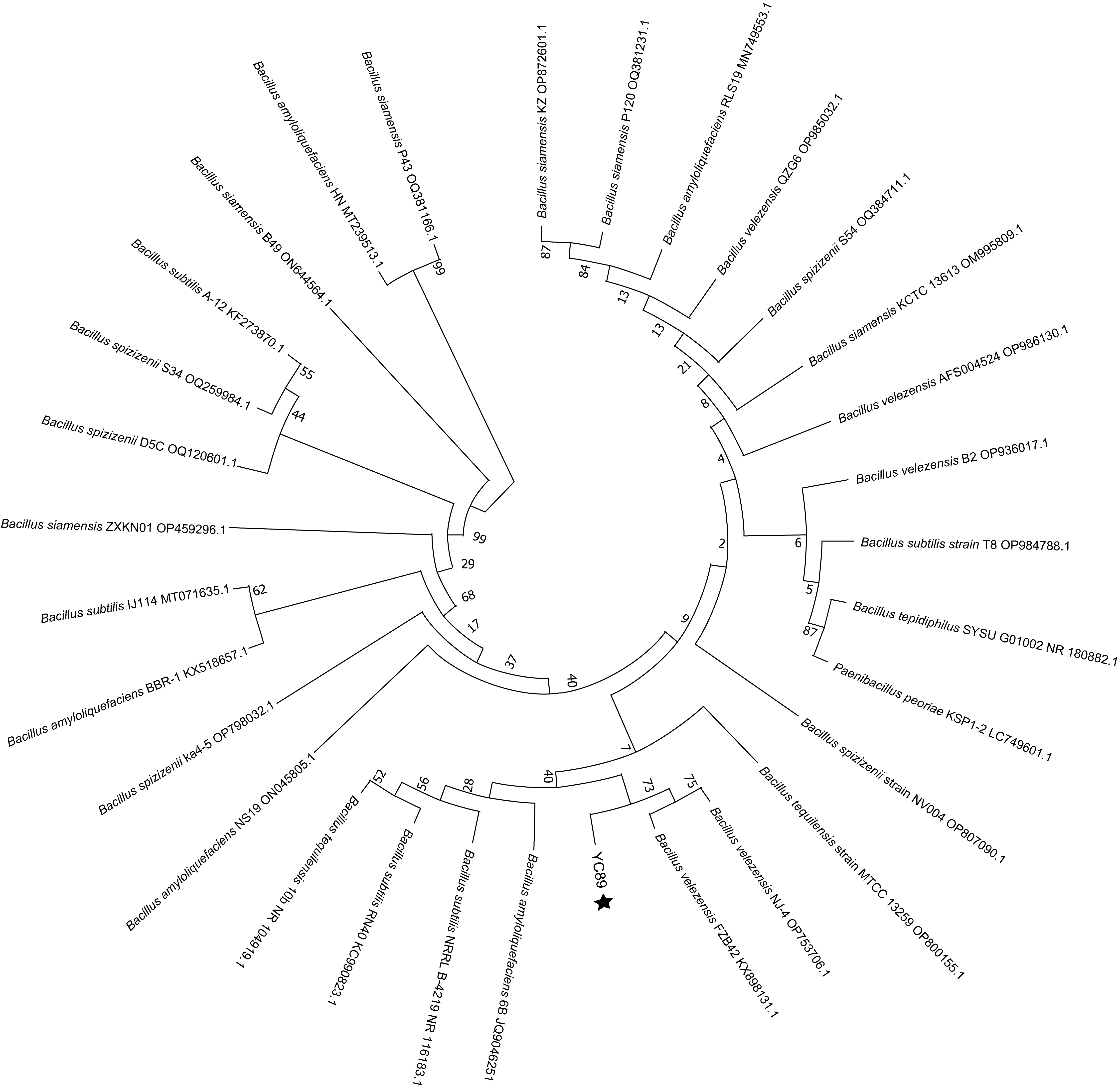


**Supplementary Figure S3 A :** Phylogenetic tree construction of YC89 strain based on 16S rRNA.

**
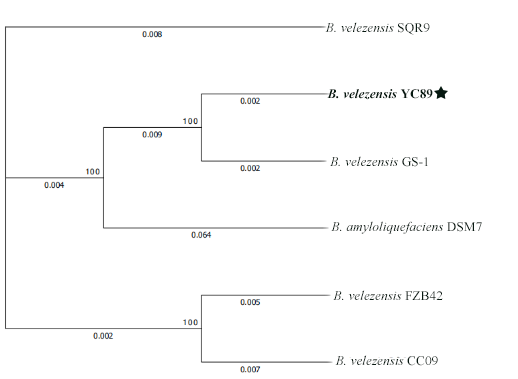
**

**Supplementary Figure S3 B:**  The tree was generated based on the single-copy core genes .


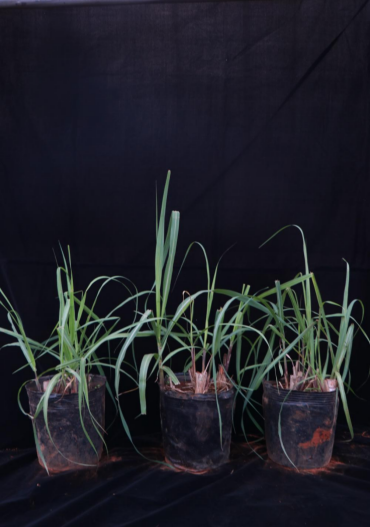


**Supplementary Figure S4 A:** Greenhouse pot experiment of CK


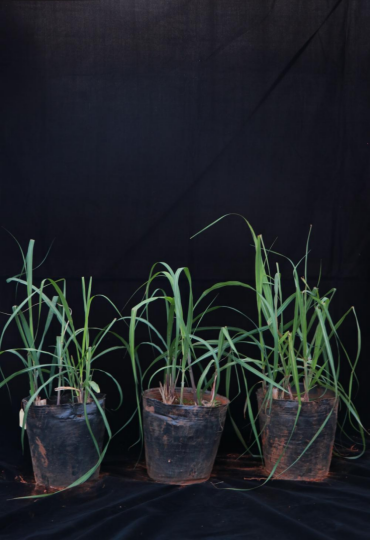


**Supplementary Figure S4 B:** Greenhouse pot experiment of Carbendazim


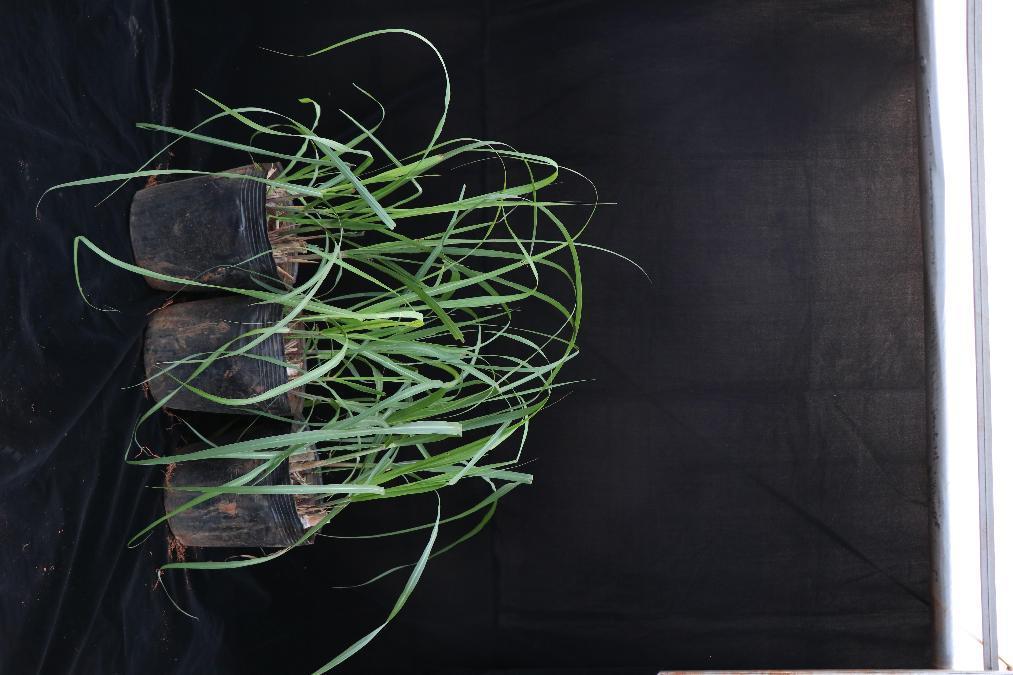


**Supplementary Figure S4 C:** Greenhouse pot experiment of X22


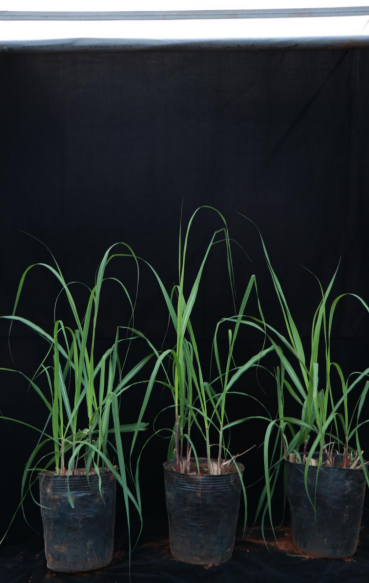


**Supplementary Figure S4 D:** Greenhouse pot experiment of YC89 + X22


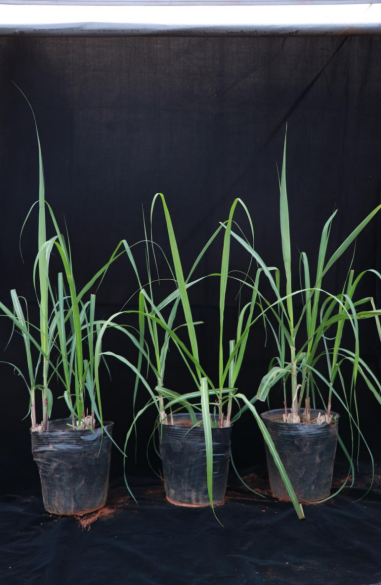


**Supplementary Figure S4 E:** Greenhouse pot experiment of YC89

## Supplementary Tables

**Supplementary Table S1:** Basic information of the reference strain

| Strains | Genbank | Size (Mb) | GC (%) | Source |
| --- | --- | --- | --- | --- |
| *Bacillus velezensis* FZB42 | NC_009725.2 | 3.9186 | 46.5 | Beet rhizosphere |
| *Bacillus velezensis* SQR9 | NZ_CP006890.1 | 4.1170 | 46.1 | Cucumber rhizosphere |
| *Bacillus velezensis* CC09 | NZ_CP015443.1 | 4.1671 | 46.1 | healthy leaves of *Cinnamomum camphora* |
| *Bacillus velezensis* GS-1 | NZ_CP072791.1 | 4.0308 | 47.06 | rhizosphere soil of ginseng |
| *Bacillus amyloliquefaciens* DSM7 | NC_014551.1 | 3.9802 | 46.1 | Soil, fermentation plant |

**Supplementary Table S2:** *B. velezensis* YC89 GO Comment Result (Top 10 Lines)

| GO ID (level1) | GO Term (level1) | GO ID (level2) | GO Term (level2) | number_of_YC89 (All) |
| --- | --- | --- | --- | --- |
| GO:0008150 | Biological Process | GO:0009987 | cellular process | 2708 |
| GO:0008150 | Biological Process | GO:0044699 | single-organism process | 2483 |
| GO:0008150 | Biological Process | GO:0008152 | metabolic process | 2393 |
| GO:0008150 | Biological Process | GO:0051179 | localization | 878 |
| GO:0008150 | Biological Process | GO:0050896 | response to stimulus | 851 |
| GO:0008150 | Biological Process | GO:0065007 | biological regulation | 792 |
| GO:0008150 | Biological Process | GO:0071840 | cellular component organization or biogenesis | 767 |
| GO:0008150 | Biological Process | GO:0050789 | regulation of biological process | 717 |
| GO:0008150 | Biological Process | GO:0032502 | developmental process | 469 |
| GO:0008150 | Biological Process | GO:0000003 | reproduction | 276 |

**Supplementary Table S3:** 42 unique CDS of YC89 strain

| ID | Product | |
| --- | --- | --- |
| MJ920_01805 | condensation domain-containing protein | |
| MJ920_03600 | hypothetical protein | |
| MJ920_03745 | collagen-like protein | |
| MJ920_04410 | hypothetical protein | |
| MJ920_04470 | hypothetical protein | |
| MJ920_04475 | hypothetical protein | |
| MJ920_06385 | ATP-binding cassette domain-containing protein | |
| MJ920_06390 | ABC transporter permease | |
| MJ920_06400 | sensor histidine kinase | |
| MJ920_06405 | response regulator transcription factor | |
| MJ920_06410 | plantaricin C family lantibiotic | |
| MJ920_06415 | type 2 lantipeptide synthetase LanM family protein | |
| MJ920_06420 | peptidase domain-containing ABC transporter | |
| MJ920_06425 | class II lanthipeptide, LchA2/BrtA2 family | |
| MJ920_06430 | class II lanthipeptide, LchA2/BrtA2 family | |
| MJ920_06435 | type 2 lantipeptide synthetase LanM family protein | |
| MJ920_07115 | DUF4917 family protein | |
| MJ920_09330 | hypothetical protein | |
| MJ920_09695 | hypothetical protein | |
| MJ920_09720 | hypothetical protein | |
| MJ920_09725 | DNA cytosine methyltransferase | |
| MJ920_10335 | YjcZ family sporulation protein |  |
| MJ920_10430 | hypothetical protein |  |
| MJ920_10450 | GNAT family N-acetyltransferase |  |
| MJ920_11595 | cytochrome P450 |  |
| MJ920_13970 | hypothetical protein |  |
| MJ920_15235 | MerR family transcriptional regulator |  |
| MJ920_15375 | hypothetical protein |  |
| MJ920_17505 | hypothetical protein |  |
| MJ920_18230 | ABC transporter ATP-binding protein |  |
| MJ920_18235 | ABC transporter permease |  |
| MJ920_18240 | ABC transporter permease |  |
| MJ920_18245 | hypothetical protein |  |
| MJ920_18725 | hypothetical protein |  |
| MJ920_18730 | hypothetical protein |  |
| MJ920_18775 | DUF262 domain-containing protein |  |
| MJ920_18780 | FRG domain-containing protein |  |
| MJ920_19095 | RES family NAD+ phosphorylase |  |
| MJ920_19100 | sce7725 family protein |  |
| MJ920_19105 | sce7726 family protein |  |
| MJ920_19110 | hypothetical protein |  |
| MJ920_19460 | restriction endonuclease |  |

**Supplementary Table S4:** YC89 CAZyme class

| family | count | family | count | family | count | family | count |
| --- | --- | --- | --- | --- | --- | --- | --- |
| AA1 | 1 | GH0 | 12 | GH3 | 8 | GH99 | 1 |
| AA10 | 1 | GH1 | 20 | GH30_3 | 1 | GT0 | 16 |
| AA4 | 1 | GH10 | 2 | GH30_8 | 1 | GT1 | 11 |
| AA7 | 2 | GH101 | 2 | GH32 | 12 | GT13 | 2 |
| CBM12 | 2 | GH109 | 1 | GH33 | 1 | GT2 | 108 |
| CBM13 | 4 | GH11 | 2 | GH35 | 1 | GT20 | 1 |
| CBM16 | 1 | GH126 | 1 | GH36 | 2 | GT24 | 1 |
| CBM2 | 1 | GH13 | 2 | GH37 | 4 | GT26 | 1 |
| CBM22 | 3 | GH130 | 1 | GH39 | 2 | GT27 | 1 |
| CBM23 | 1 | GH133 | 1 | GH4 | 11 | GT28 | 7 |
| CBM26 | 2 | GH135 | 1 | GH43_11 | 3 | GT29 | 1 |
| CBM3 | 2 | GH13_11 | 8 | GH43_16 | 1 | GT30 | 1 |
| CBM38 | 1 | GH13_26 | 1 | GH43_24 | 2 | GT32 | 1 |
| CBM48 | 8 | GH13_28 | 2 | GH43_4 | 1 | GT4 | 62 |
| CBM5 | 7 | GH13_29 | 2 | GH43_5 | 1 | GT47 | 1 |
| CBM50 | 47 | GH13_3 | 10 | GH46 | 1 | GT49 | 1 |
| CBM54 | 2 | GH13_30 | 1 | GH5 | 1 | GT5 | 1 |
| CBM57 | 1 | GH13_31 | 2 | GH51 | 3 | GT51 | 12 |
| CBM6 | 2 | GH13_32 | 1 | GH53 | 3 | GT57 | 1 |
| CBM66 | 3 | GH16 | 4 | GH5_11 | 2 | GT61 | 1 |
| CE0 | 1 | GH17 | 2 | GH5_2 | 1 | GT66 | 1 |
| CE1 | 7 | GH18 | 3 | GH6 | 2 | GT68 | 6 |
| CE11 | 6 | GH19 | 4 | GH65 | 2 | GT7 | 1 |
| CE12 | 3 | GH2 | 9 | GH68 | 1 | GT8 | 4 |
| CE14 | 8 | GH23 | 13 | GH72 | 1 | GT83 | 1 |
| CE4 | 13 | GH24 | 1 | GH73 | 8 | GT9 | 6 |
| CE6 | 2 | GH25 | 1 | GH76 | 1 | GT96 | 1 |
| CE7 | 1 | GH26 | 1 | GH92 | 1 | PL1_6 | 1 |
| CE9 | 5 | GH28 | 3 | GH93 | 3 | PL1_8 | 1 |
